# Single-cell transcriptome identifies *FCGR3B* upregulated subtype of alveolar macrophages in patients with critical COVID-19

**DOI:** 10.1016/j.isci.2021.103030

**Published:** 2021-08-25

**Authors:** Nasna Nassir, Richa Tambi, Asma Bankapur, Saba Al Heialy, Noushad Karuvantevida, Hamda Hassan Khansaheb, Binte Zehra, Ghausia Begum, Reem Abdel Hameid, Awab Ahmed, Zulfa Deesi, Abdulmajeed Alkhajeh, K.M. Furkan Uddin, Hosneara Akter, Seyed Ali Safizadeh Shabestari, Omar Almidani, Amirul Islam, Mellissa Gaudet, Richard Kumaran Kandasamy, Tom Loney, Ahmad Abou Tayoun, Norbert Nowotny, Marc Woodbury-Smith, Proton Rahman, Wolfgang M. Kuebler, Mahmood Yaseen Hachim, Jean-Laurent Casanova, Bakhrom K. Berdiev, Alawi Alsheikh-Ali, Mohammed Uddin

**Affiliations:** 1 College of Medicine, Mohammed Bin Rashid University of Medicine and Health Sciences, Dubai, UAE; 2 Meakins-Christie Laboratories, Research Institute of the McGill University Health Center, Montreal, QC, Canada; 3 Dubai Health Authority, Microbiology and Infection Control Unit, Pathology and Genetics Department, Latifa Women and Children Hospital, Dubai, UAE; 4 Medical Education & Research Department, Dubai Health Authority, Dubai, UAE; 5 Genetics and Genomic Medicine Centre, NeuroGen Children’s Healthcare, Dhaka, Bangladesh; 6 Nuffield Department of Surgical Science, University of Oxford, Oxford, UK; 7 Cellular Intelligence (Ci) Lab, GenomeArc Inc., Toronto, ON, Canada; 8 Al Jalila Genomics Center, Al Jalila Children’s Hospital, Dubai, UAE; 9 Institute of Virology, University of Veterinary Medicine Vienna, Vienna, Austria; 10 Biosciences Institute, Newcastle University, Newcastle Upon Tyne, UK; 11 Department of Rheumatology, Memorial University of Newfoundland, St Johns, NL, Canada; 12 Institute of Physiology, Charité - Universitätsmedizin Berlin, Berlin Germany; 13 St. Giles Laboratory of Human Genetics of Infectious Diseases, Rockefeller Branch, The Rockefeller University, New York, NY, USA; 14 Laboratory of Human Genetics of Infectious Diseases, Necker Branch, INSERM U1163, Paris, France; 15 University of Paris, Imagine Institute, Paris, France; 16 Howard Hughes Medical Institute, New York, NY, USA; 17 Dubai Health Authority, Dubai, UAE

**Keywords:** molecular biology, transcriptomics, virology

## Abstract

Understanding host cell heterogeneity is critical for unraveling disease mechanism. Utilizing large-scale single-cell transcriptomics, we analyzed multiple tissue specimens from patients with life-threatening COVID-19 pneumonia, compared with healthy controls. We identified a subtype of monocyte-derived alveolar macrophages (MoAMs) where genes associated with severe COVID-19 comorbidities are significantly upregulated in bronchoalveolar lavage fluid of critical cases. *FCGR3B* consistently demarcated MoAM subset in different samples from severe COVID-19 cohorts and in *CCL3L1*-upregulated cells from nasopharyngeal swabs. *In silico* findings were validated by upregulation of *FCGR3B* in nasopharyngeal swabs of severe ICU COVID-19 cases, particularly in older patients and those with comorbidities. Additional lines of evidence from transcriptomic data and *in vivo* of severe COVID-19 cases suggest that *FCGR3B* may identify a specific subtype of MoAM in patients with severe COVID-19 that may present a novel biomarker for screening and prognosis, as well as a potential therapeutic target.

## Highlights

• Association of MoAM subtype with severe COVID-19 cases presented with comorbidities
• Upregulated *FCGR3B* in *CCL3L1* positive MoAM cells in severe COVID-19 cases
• Upregulated *FCGR3B* within MoAM subtype as a potential marker for COVID-19 severity

## Graphical abstract

**Figure:**
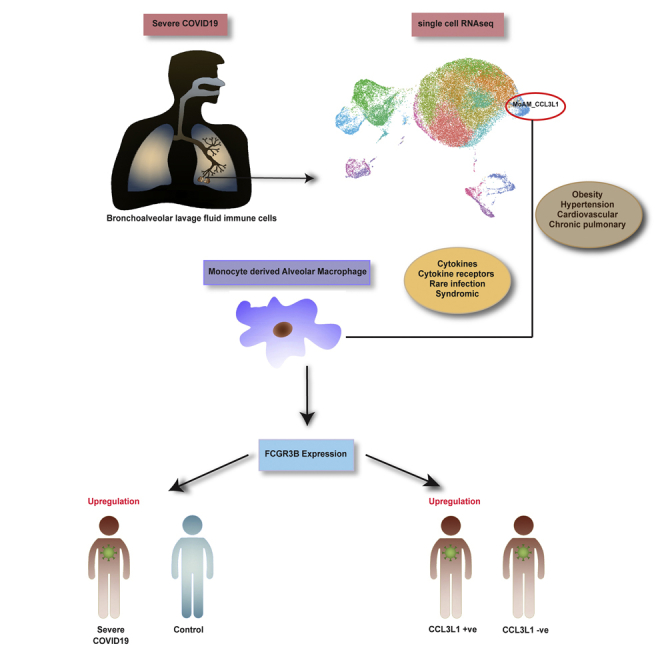

## Introduction

Although most individuals infected with SARS-CoV-2 remain asymptomatic or develop mild forms of the disease, a significant proportion (approximately 5%) of infected cases develops severe COVID-19 pneumonia and a smaller proportion (0.5%) critical pneumonia (Hu et al., 2020; Uddin et al., 2020). The major predictor of critical COVID-19 disease is age >60 years, while comorbidities such as obesity, diabetes mellitus, hypertension, cardiovascular disease, and chronic pulmonary disease play a modest role (Sanyaolu et al., 2020). A small fraction (<0.01%) develop an inflammatory syndrome closely resembling Kawasaki disease, which is now designated as multisystem inflammatory syndrome in children (Kabeerdoss et al., 2021). Similarly, a multitude of additional factors including sex, blood group, etc. may impact on the clinical manifestations of COVID-19 following respiratory infection with SARS-CoV-2, thus complicating the identification and prognosis of a common molecular pathogenic mechanism.

Major international initiatives are underway to unravel the pathogenesis of severe COVID-19. Despite these efforts, our knowledge of COVID-19 pathophysiology is very limited and has been compounded by the complex interplay with a range of comorbid conditions. Recently, rare germline mutations impairing TLR3 and IRF7 dependent type I interferon immunity were found to be causal for a minor subset of patients with critical COVID-19 (Bastard et al., 2020; Zhang et al., 2020d). At least 10% of critical COVID-19 cases can be explained by the presence of their autoimmune phenocopy, in the form of neutralizing autoantibodies to type I IFNs (Bastard et al., 2020; Zhang et al., 2020c, 2020d). From the international COVID-19 Host Genetics Initiative, thirteen loci have been found to be associated with severe COVID-19, with OR < 2, and were subsequently replicated in other independent population cohorts (Initiative, 2021; Severe Covid et al., 2020). Yet, these signals fail to explain the pathophysiology of most of the critical COVID-19 cases (Hameid et al., 2021), demonstrating that the severity and progression of the disease is largely determined by acquired regulatory factors impacting the individual patients host response.

The transcriptional signature in severe COVID-19 provides insight into this host response by identification of differentially expressed genes enriched in specific key pathophysiological pathways. Traditionally, studies have performed RNA-seq analyses on bulk tissue samples. While this approach yields important information in homogeneous cell samples, data become increasingly difficult to interpret with complexity of multicellular organs or samples such as lung or blood. More recently, this limitation has been overcome by single-cell RNA sequencing which has been successfully utilized to analyze transcriptomic profiles in heterogeneous cell samples of patients with severe COVID-19, including blood peripheral mononuclear cells (PBMCs), bronchoalveolar lavage fluid (BALF), and lung and airway epithelium (Aschenbrenner et al., 2021; Chua et al., 2020; Schulte-Schrepping et al., 2020). Collectively, these studies identified specific cell types that are associated with or contribute to the development of critical COVID-19. Identification of specific cellular signatures not only provides potential insight into the pathogenic mechanisms of severe COVID-19 but also yields important biomarkers for prognostic screening and personalized clinical management, as early detection of patients at risk and escalation of medical management may help prevent serious complications. Although several biological markers associated with immune response in COVID-19 have been reported (Caruso et al., 2020; Laing et al., 2020; Malik et al., 2020), there remains a paucity of markers that would allow the stratification of COVID-19 according to severity. We hypothesize that SARS-CoV-2 infection activates certain “cell type” with identifiable gene regulatory signature that are strongly associated with severe COVID-19 phenotypes (i.e., age, comorbidities). Our study incorporating single-cell analysis and clinical data demonstrates the association between specific cell types, modulator genes, and COVID-19 severity with age and comorbidities.

## Results

### Single-cell transcriptomics and cell type characterization

**Figure 1:**
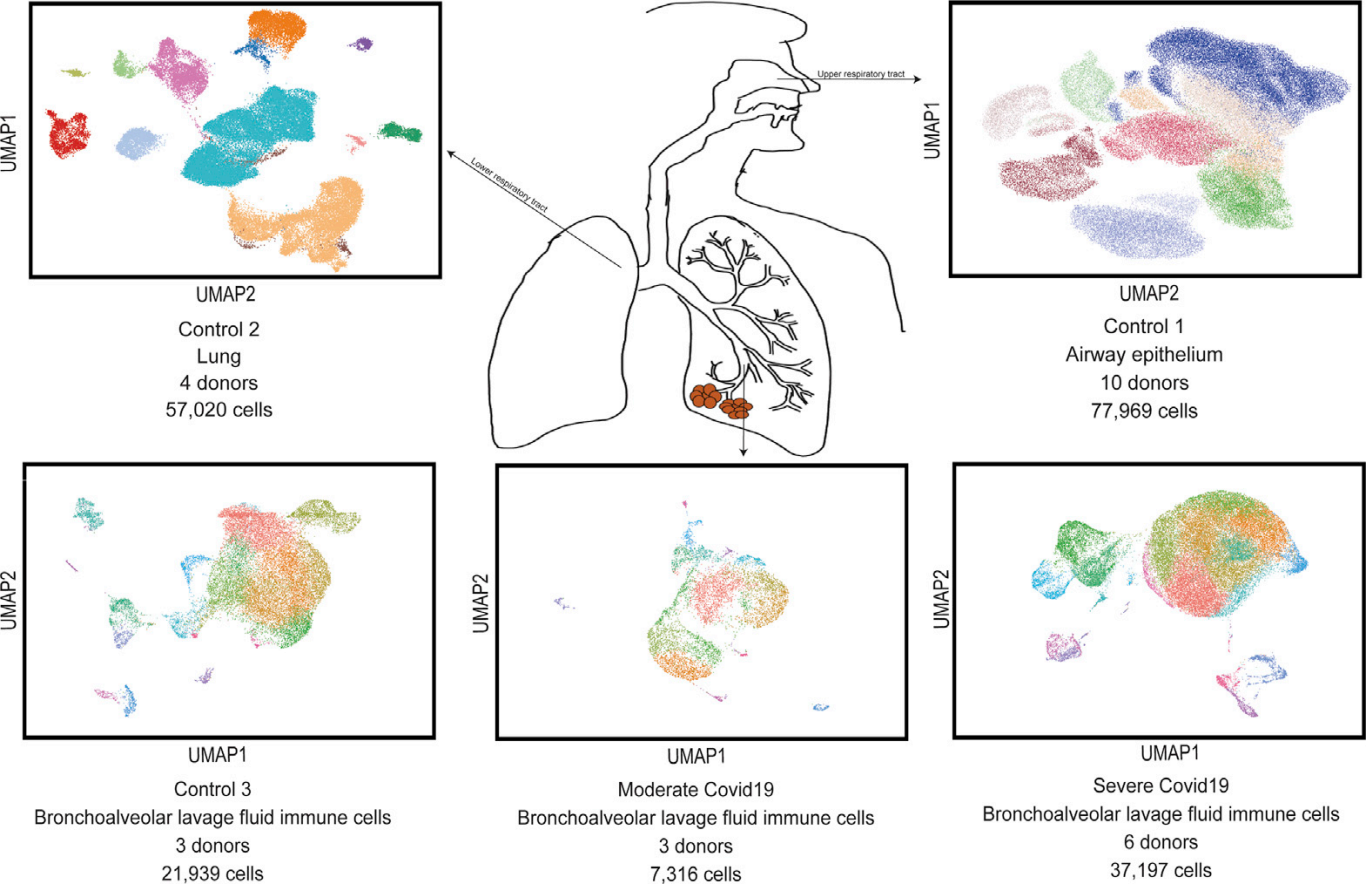
Single-cell transcriptomics data Construction of cell clusters from single-cell RNA-seq derived from upper (airway epithelium) and lower respiratory tract (lung) in healthy controls, bronchoalveolar lavage fluid (BALF) within control, moderate and severe COVID-19 donors. Clustering of single-cell transcriptome data shown is defined by the uniform manifold approximation and projection (UMAP) plot and each color represents a unique cluster. Tissue region from where samples were collected, number of donors and number of cells are depicted in the figure.

To identify cell types associated with severe COVID-19, multiple single-cell transcriptomics data sets were used. Control data sets 1 and 2 from healthy airways of 10 volunteers and healthy lungs of 4 donors, respectively, were recreated from Deprez et al. (2020) and Madissoon et al. (2019) (Figures 1 and S1). We further used 14 samples of BALF single-cell data (Liao et al., 2020), segregated them into control, moderate, and severe COVID-19 samples and generated individual single-cell maps for each group. Using the signature marker gene list from Liao *et al.*(2020), these recapitulated clusters from all combined samples were largely defined as macrophages, myeloid dendritic cells, and T-cells; however, a major part of these clusters remained ambiguous (Figure S2). We re-computed and identified a total of 19, 17, and 18 sets of clusters for control, moderate, and severe samples consisting of 21,939, 7316, and 37,197 cells, respectively (Figure 1).

**Figure 2:**
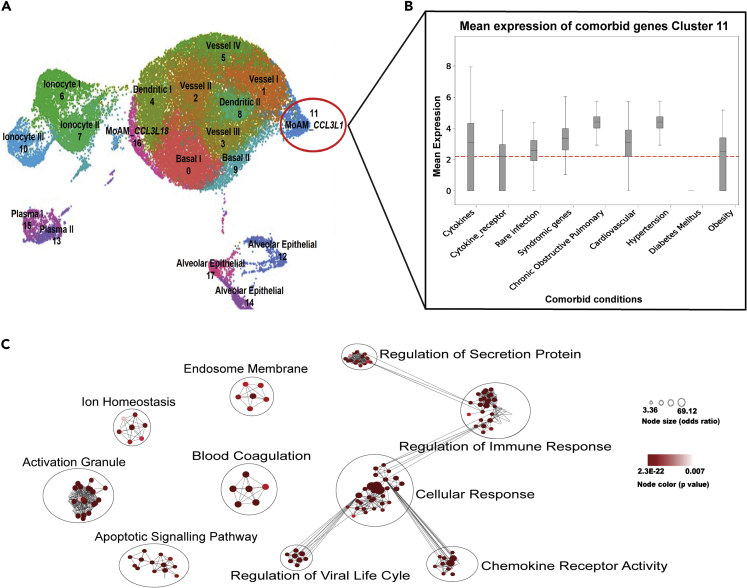
Cluster identity and classification (A) Severe COVID-19 cluster annotated using signature marker gene list. (B) The mean expression (y axis) of genes that are associated with comorbid diseases with severe COVID-19 (x axis) of MoAM_*CCL3L1* (circled red) cell type within severe COVID-19 bronchoalveolar lavage fluid (BALF) shown in box plot. 99th percentile expression value from the severe dataset is marked using red dotted lines. Y axis represent the mean of log normalized expression value for comorbid genes across the cell clusters. (C) Pathway analysis of MoAM_*CCL3L1* cluster drawn using Cytoscape. Color gradient and size of nodes represents p value and Odds ratio, respectively.

To assign the corresponding cell type identity to each cluster, we utilized an in-house database (Table S1) of specific markers of lung tissue cell types that was created using a combination of literature search (Deprez et al., 2020; Grant et al., 2021; Madissoon et al., 2019; Reyfman et al., 2019) and cell marker databases (Franzen et al., 2019; Zhang et al., 2019). A specific cell type was defined for a particular cluster if it had a distinct higher normalized expression (by comparison of medians) for the respective signature marker genes from the database (Figure S3). Overall, the clusters of severe COVID-19 samples were constituted by basal cells, vascular cells, dendritic cells, ionocytes, monocyte-derived alveolar macrophages, plasma cells, and alveolar epithelial cells (Figure 2A). Clusters in control and moderate COVID-19 samples were assigned analogously (Table S2; Figure S4).

### Comorbid gene set upregulation in macrophage subtype cluster 11

We have used genes that are associated with severe COVID-19 comorbid conditions to identify specific activated cell types that are highly regulated in severe COVID-19 phenotypes. The list included encoding cytokines and cytokine receptors, or associated with rare infectious diseases, rare syndromes, chronic obstructive pulmonary disease, cardiovascular disease, hypertension, obesity, and diabetes (Table S3). The enrichment analysis of cluster genes with comorbid gene lists revealed that eight of the nine auxiliary COVID-19 comorbid condition genes were exclusively upregulated in cluster 11 in severe COVID samples (Figures 2B and S5). Severe cluster 11 was marked as monocyte-derived alveolar macrophages (MoAMs), as indicated by the presence of *CCl3L1* (Figure 2A). This particular MoAM subtype was not found in any of the moderate or control clusters.

### Macrophage cell subtype-associated pathways

To further characterize the MoAM cell subtype, the functional enrichment of DE genes in specific biological pathways was examined using Kyoto Encyclopedia of Genes and Genomes (KEGG) and Gene Ontology (GO) gene sets (Table S4). These analyses revealed that the identified MoAM cell subtype is involved in host immune response signaling networks related to TNFα (p < 2.79 × 10^−25^), cytokine and interferon gamma responses (p < 1.80 × 10^−22^), the response to type1 interferon and biotic stimulus (p < 2.75 × 10^−17^), and innate immune and inflammatory responses (p < 1.09 × 10^−16^), which were visualized using Cytoscape (Figure 2C).

### *FCGR3B-*restricted expression in MoAM cell subtype of severe COVID-19 cluster

**Figure 3:**
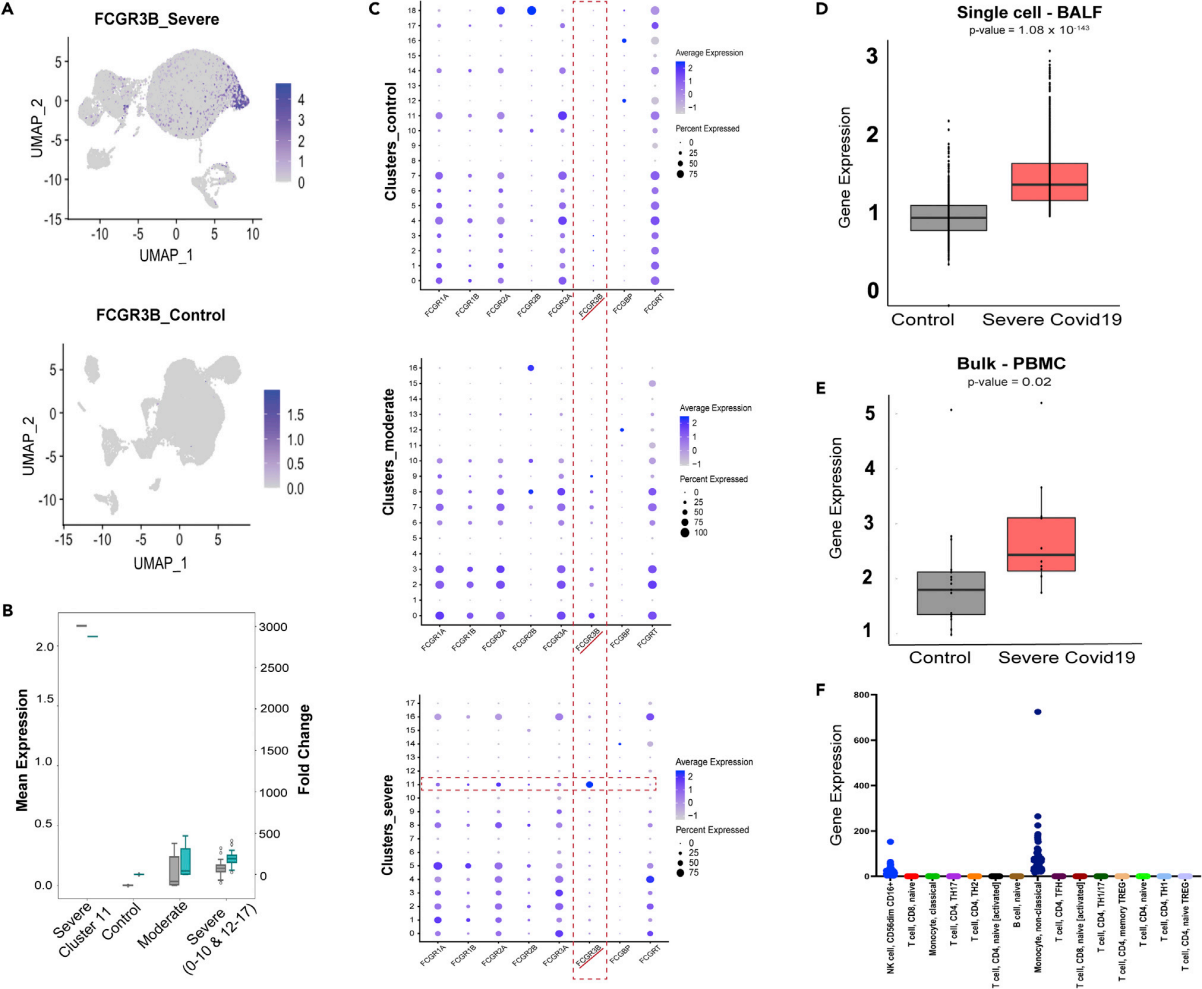
Cell type specific marker characterization (A) Feature plot showing restricted expression of *FCGR3B* in severe cluster 11 and not in control. (B) Boxplot showing higher mean expression (gray) and fold change (blue) of *FCGR3B* gene in severe cluster 11 compared to control, moderate and other severe clusters. Y axis represents the mean of log normalized expression value for each gene across the cells. (C) Dotplot showing expression of the FcR genes (FCGR1A, FCGR1B, FCGR2A, FCGR2B, FCGR3A - Fc Fragment Of IgG Receptor Ia, Ib, IIa, IIb, IIIa respectively, FCGBP - Fc Fragment Of IgG Binding Protein, FCGRT - Fc Fragment Of IgG Receptor And Transporter) in the control, moderate and severe clusters. (D) Validation of expression of *FCGR3B* in severe COVID-19 (red) and control samples (gray) using single-cell BALF data. The y axis represents the log normalized expression value calculated using Seurat. Expression counts per cell were divided by the total counts for that cell (sequencing depth) and multiplied by the 10,000 (scale factor), which was then log transformed. (E) Validation of expression of *FCGR3B* in severe COVID-19 (red) and control samples (gray) using bulk PBMC data. (F) Dotplot showing higher restricted expression of *FCGR3B* in non-classical monocytes of normal immune cells as extracted from RNAseq of DICE (Database of Immune Cell Expression, Expression quantitative trait loci (eQTLs) and Epigenomics).

Since severe cluster 11 (or MoAM cell subtype) was enriched with both COVID-19 comorbid condition genes and immune response pathways, the signature genes for this specific cell type were analyzed in detail. Primarily, we selected the top 20 genes from cluster 11 based on significant expression fold change defining the marked difference of relative expression of genes with respect to other clusters for samples from control, moderate, and severe patients (Figure S6). We further analyzed the expression of these individual genes across all the clusters of control data set 1, control data set 2, control data set 3, moderate and severe data sets both qualitatively (Figure S7) and quantitatively (Figures S8 and S9). We then selected the genes that were exclusively enriched in cluster 11 of the severe data set and minimally expressed in all other clusters of severe, moderate, and control patients. By this approach, we identified the most differentially regulated gene that demarcated cluster 11 in samples from patients with severe COVID-19, namely *FCGR3B* (Figures 3A and 3B). FcRs regulate both adaptive and innate immune responses which are crucial for the defense against infection and prevention of chronic inflammation or autoimmune diseases. Upon crosslinking by immune complexes, FcRs mediate important immune responses such as release of cytokines or phagocytosis (Ben Mkaddem et al., 2019). Expression of FcR genes in clusters of control, moderate, and severe data sets were monitored, and we found selective upregulation in severe cluster 11 to be specific for *FCGR3B* as compared with other FcR (CD16 encoding) genes (Figure 3C). The expression of *FCGR3B* was then validated in another single-cell BALF data set (Grant et al., 2021) in which upregulation of *FCGR3B* in severe COVID-19 samples as compared with control was again evident (Figure 3D, p < 1.08 × 10^−143^). We then assessed the expression of *FCGR3B* in bulk PBMC data (Arunachalam et al., 2020) (Figure 3E, p = 0.02) and found *FCGR3B* expression to be restricted to non-classical monocytes in multiple blood single-cell data sets (Can Liu et al., 2021; Schmiedel et al., 2018) (Figures 3F and S10).

**Figure 4:**
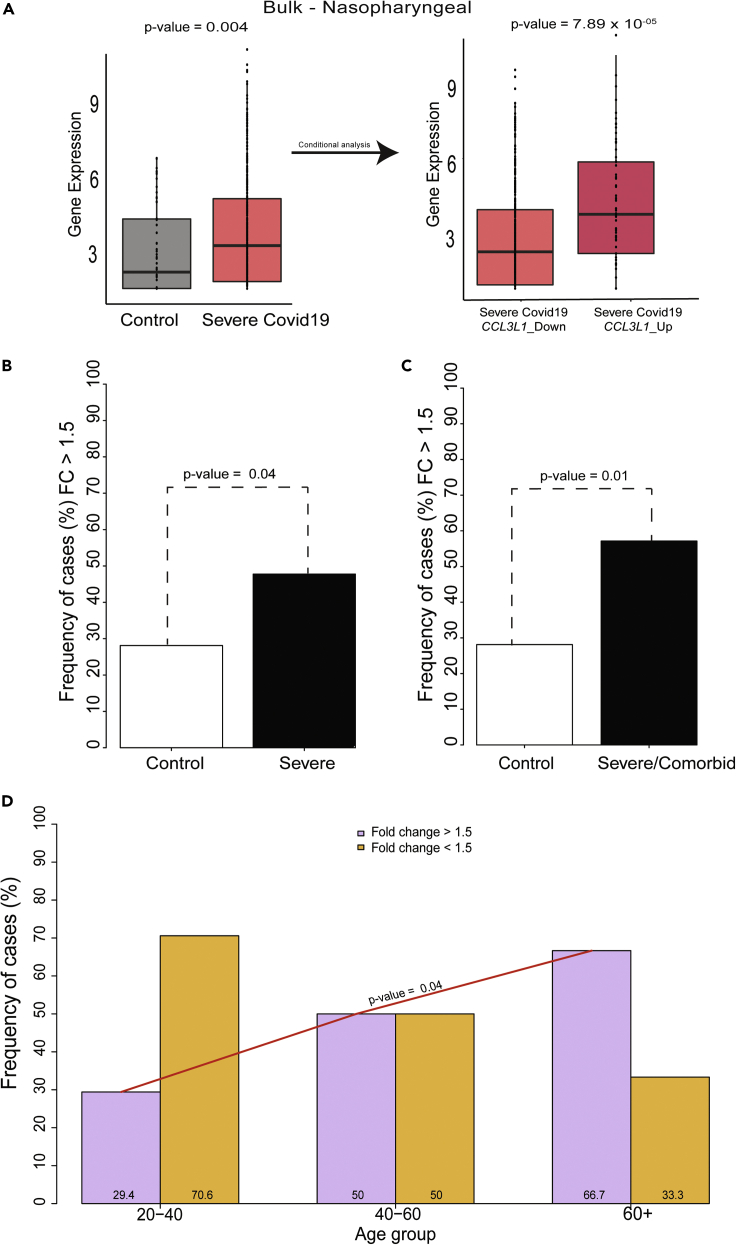
*FCGR3B* as a modulator of COVID-19 severity (A) Validation of expression of *FCGR3B* in severe COVID-19 (red) and control samples (gray) using bulk nasopharyngeal data. Expression of *FCGR3B* in severe COVID-19 samples having *CCL3L1* downregulated and upregulated expression. For the bulk RNA seq data, y axis represents the log transformed count per million expression value computed using EdgeR package. (B) Fraction of samples with fold change of *FCGR3B* greater than 1.5 in severe COVID-19 patients and control. (C) Fraction of samples with fold change of *FCGR3B* greater than 1.5 in severe COVID-19 patients with comorbidity and control. (D) Fraction of samples with fold change of *FCGR3B* greater than 1.5 across different age group.

We further validated the expression of *FCGR3B* in a bulk data set from nasopharyngeal swabs and observed upregulation of *FCGR3B* in severe COVID-19 samples compared with control (Figure 4A, p = 0.004). *CCL3L1-CCR5* signaling is important in several inflammatory responses, including macrophage function, and T-cell-dependent immune responses (Menten et al., 2002) and *CCL3L1* is a specific marker for MoAM (Grant et al., 2021). We thus looked at the expression of *FCGR3B* in severe cases with upregulated expression of *CCL3L1* in bulk nasopharyngeal data (Figure 4A, p = 7.89 × 10^−5^) compared with severe cases with low *CCL3L1* expression. Similar analysis was performed for another candidate gene, *FFAR2*, exclusively expressed in severe cluster 11 (Figure S11). Additionally, elevated levels of TNF-ɑ have been reported in COVID-19 patient samples (Del Valle et al., 2020; Feldmann et al., 2020) and TNF-α is considered an indicator of severity in COVID. As *TNFAIP6* was also restrictively expressed in severe cluster 11, similar analysis was performed. Yet, in contrast to *FCGR3B,* the expression of *TNFAIP6* (indicator of COVID-19 severity) and *CCL3L1* (marker for MoAM) is not restricted to one cluster in all three datasets (Figures S8, S9 and S12).

### *FCGR3B* upregulation in severe COVID-19 clinical samples

To test whether *FCGR3B* can be used as a marker for COVID-19 severity, we used clinical samples of severe COVID-19 patients obtained from Dubai Health Authority. The nasopharyngeal swabs of critical (hospitalized in intensive care unit (ICU)) qPCR positive COVID-19 patients (n = 31) and qPCR negative controls (n = 11) were subjected to RNA isolation and qPCR analysis for *FCGR3B* and *FFAR2*. Among patients with severe COVID-19, 50% had greater than 1.5-fold change of *FCGR3B* compared with 28.1% in controls (Figure 4B). Moreover, 57.1% of patients with severe COVID-19 with comorbidity had greater than 1.5-fold change of *FCGR3B* compared with 28.1% in controls (Figure 4C, p = 0.01, Figure S13; Table S5). Further, among patients with COVID-19, the frequency of cases with fold change of *FCGR3B* greater than 1.5 was significantly higher among patients older than 60 years compared with younger patients (20–40 years) (Figure 4D, p = 0.04). Similar analysis was performed for *FFAR2* gene (Figure S14), which did not however show any significant association with COVID-19 severity.

### *In vitro* upregulation of *FCGR3B* in spike protein stimulated obese subjects

Since severity of COVID-19 is associated with obesity (Stefan et al., 2021), we performed *in vitro* experiments on tissue samples from obese patients. Normal human primary bronchial epithelial (NHBE) cells were obtained from non-obese and obese subjects and stimulated with 1 μg/mL of SARS-CoV-2 spike protein. Conditioned media were harvested, added to cultured monocytes, and incubated overnight. The cells were pelleted for RNA extraction, subjected to reverse transcription, and mRNA expression of *FCGR3B* and *GAPDH* (as housekeeping gene) was measured. The expression of *FCGR3B* in monocytes treated with conditioned medium from spike protein stimulated NHBE of obese subjects was significantly higher than in non-obese subjects (p = 0.006), suggesting *FCGR3B* as a potential modulator of COVID-19 severity in patients with obesity (Figure S15, Tables S6, S7, S8).

## Discussion

Severe COVID-19 manifests primarily as a respiratory disease but may involve multiple systemic organs as the disease progresses. Specific cell types and major regulatory genes that confer progression to critical illness in some patients following SARS-CoV-2 infection are, however, poorly understood. SARS-CoV-2 primarily infects the respiratory epithelium, and although the presence of viral components in blood or systemic organs has occasionally been documented, it seems that disease progression in both lungs and systemic organs is largely governed by a dysregulation of inflammatory and pro-coagulatory responses rather than viral infection of the respective organ *per se*. Previous single-cell transcriptome analyses (Aschenbrenner et al., 2021; Chua et al., 2020; Schulte-Schrepping et al., 2020) reported differentially regulated cell types that lacks the connection of severe COVID-19 phenotypic indicators (i.e., age, comorbidities). In this study, we have identified a subtype of macrophage where regulatory genes are associated with severe COVID-19 phenotypic indicators.

BALF has been widely used for diagnosis and study of lower respiratory airway infections (Kahn and Jones, 1987) including SARS-CoV-2 (Liao et al., 2020), due to its limited invasiveness and ability to reflect pathophysiological processes in the distal airspaces. We thus analyzed cell type maps of BALF and lung at single-cell resolution to account for the dynamic nature of cellular responses by numerous cell types and identify modulators specific and potentially critical for the development from SARS-CoV-2 infection to severe COVID-19. Host immune response is our primary defense against acute COVID-19 infection, yet if dysregulated may also lead to simultaneous injury of host organs and tissues. Tissue injury because of an uncontrolled inflammatory response is the main driver for lung injury in patients with severe COVID-19, which often develops and persists long after the virus has already been eliminated, and determines disease severity, progression, and outcome (Zhang et al., 2020b). Since the onset of the COVID-19 pandemic, various adaptive and innate immune cells have been implicated in this dysregulated immune response including T cells, B cells, neutrophils, as well as MoAMs (Hosseini et al., 2020; Zhou et al., 2020a). Importantly, recruitment of MoAM into the lungs has recently been documented in African green monkeys infected with SARS-CoV-2 (Hartman et al., 2020). Besides, it was also demonstrated that SARS-CoV2 abortively infects the monocyte-derived macrophages and dendritic cells causing cytokine and chemokine storm and type I IFN–mediated cell death (Zheng et al., 2021). In the present study, we identified a *FCGR3B* upregulated alveolar macrophage subtype that was derived from recruited monocytes. We have identified an abundance of this *FCGR3B* upregulated monocyte-derived alveolar macrophage cell population (cluster 11 marked by *CCL3L1*) in multiple tissue data sets of respiratory samples from severe COVID-19 single-cell and bulk transcriptomes, while this cell population was absent in moderate COVID-19 and control samples (Figure S4). The expression level per cluster for *CCL3L1* and *FCGR3B* marker in severe, moderate and control single-cell transcriptome data clearly reveals a distinct pattern of *FCGR3B* and restrictive expression for MoAM (cluster 11) in severe COVID-19 cases (Figure S7; Tables S10–S12).

We have adopted an *in silico* analysis comprising of all genes known to be associated with severe COVID-19 comorbid conditions to identify this disease associated cell types. Underlying comorbidities of patients with COVID-19 including pre-existing cardiovascular conditions (Guan et al., 2020), diabetes (Bhatti et al., 2020; Zhu et al., 2020), chronic obstructive pulmonary disease (Zhao et al., 2020), and others only weakly contribute (OR <2) to the severity of infection (Wang et al., 2020). We utilized all genes associated with the aforementioned comorbidities to hone into cell types in multiple tissues. We identified a subtype of MoAM where these severe COVID-19 comorbid associated genes are highly expressed (Figure 2B). Using different transcriptomic and genomic techniques, we validated the specificity of this subtype of macrophages that is characterized by *FCGR3B* gene for severe COVD-19. Moreover, our pathway analysis revealed enrichment of the identified MoAM subtype cell population genes in inflammatory signaling networks including TNFα, cytokine and interferon gamma response, response to type1 interferon and biotic stimulus, innate immune and inflammatory response (Figure 2C). This association is particularly noteworthy since type I IFN signaling is suggested to be a key player in COVID-19, given that IFNs type I and III inhibit SARS-CoV-2 in a dose-dependent manner (Felgenhauer et al., 2020).

The restricted expression of *FCGR3B* in a comorbid gene set enriched severe COVID-19 cell type (Figure 3A) makes *FCGR3B* an interesting candidate marker gene for severe COVID-19, a notion that is further supported by the finding that *FCGR3B* was selectively expressed in severe cluster 11 compared with other FcR family genes (Figure 3C). The *FCGR3B* gene is unique amongst the FcR genes in that it encodes a GPI-anchored receptor, FcγRIIIb (CD16b). Although this receptor is typically known to be expressed on the surface of neutrophils and basophils (Simmons and Seed, 1988), recent single-cell transcriptome data suggests *FCGR3B* gene expression is significantly high in non-classical monocytes (Can Liu et al., 2021; Schmiedel et al., 2018). In the data sets analyzed in our present study, *FCGR3B* showed severe COVID-19 specific expression that was restricted to a subtype of monocyte-derived macrophages which also showed upregulation of *CCL3L1*, a known inflammatory marker for MoAMs (Tables S10–S12). This restrictive expression of *FCGR3B* was not observed in any of the control or moderate clusters (Figure S16) confirming the selective upregulation in MoAM_CCL3L1 (severe cluster 11). MoAMs are alveolar macrophages differentiated from peripheral blood monocytes (Evren et al., 2021) that are recruited to the lungs upon viral exposure. Upregulation of *FCGR3B* might shed insight into the recruitment of such monocyte as part of host response. In contrast, it has also been suggested that decreased *FCGR3B* copy number will lead to reduced immune complex clearance (Qureshi et al., 2017). Among the three monocyte subtypes, the non-classical population with low LPS co-receptor CD14 and high FCγIII receptor CD16 surface expression is found to be predominant (Sanchez-Cerrillo et al., 2020; Zhang et al., 2020a). As a result, the differentiated macrophages possess inflammatory and antigen presentation characteristics, thereby orchestrating anti-viral and fibrotic responses within the lungs. Based on the abundance of *FCGR3B* upregulated MoAMs, it is possible that dysregulated macrophage response to SARS-CoV-2 infections may promote injury to the host tissue due to macrophage activation syndrome (Merad and Martin, 2020) or serve as a Trojan horse, enabling specific viral anchoring to the pulmonary parenchyma (Abassi et al., 2020).

Upregulation of *FCGR3B* in severe COVID-19 was validated in datasets from multiple tissue types including single-cell BALF, bulk PBMC and bulk nasopharyngeal data (Figures 3D, 3E, and 4A). As *CCL3L1* is a known marker for MoAM (Grant et al., 2021) with important roles in macrophage function (Menten et al., 2002), we demonstrated *in silico* that *FCGR3B* is highly expressed in nasopharyngeal swabs within *CCL3L1* high cells compared to *CCL3L1* low cells. In addition to validating our initial findings, this transition of analysis from BALF to nasopharyngeal swabs is of potential clinical relevance given that the latter are the most available tissue type with the prospect for mass screening in the future. A cohort of severe COVID-19 patients from Dubai also exhibited a significantly higher fraction of severe patients with upregulated *FCGR3B* compared with controls (Figure 4B). This association was even more evident for severe patients with a higher burden of comorbid conditions or age (Figures 4C and 4D). Our identification of high *FCGR3B* expression in severe COVID-19 patients suggest a potential role of the corresponding protein product, FcγRIIIb, in the recruitment and activation of other immune cells to the infection site. The resulting cytokine storm may in turn promote lung injury and disseminate the course of the pathological events to other organs via systemic circulation (Quan et al., 2020). Furthermore, genomic variants, copy number variations, and single-nucleotide polymorphisms within *FCGR3B* have been shown to be significantly associated with several autoimmune diseases (Bournazos et al., 2011; McKinney and Merriman, 2012). However, conditional *FCGR3B* expression in *CCL3L1* positive isolated cells in the nasopharyngeal swabs would have precisely defined the correlation among MoAM, *FCGR3B,* and COVID-19 severity. The direct functional role of *FCGR3B* in dysregulated immunity in general, and COVID-19 specifically remains to be elucidated.

In summary, *FCGR3B* is upregulated in a macrophage subtype (MoAM_CCL3L1) that is only observed in severe COVID-19 cases, hence differential regulation due to comorbidities or genetic mutations that impair *FCGR3B* are predicted to contribute to the severe course of COVID-19. The integrated, multidimensional approach in our study lays the foundation for understanding the role of the *FCGR3B* gene positive subtype of MoAM in COVID-19 severity, which may pave the way for novel immune-targeted therapies. It will be valuable to investigate whether MoAM cells or *FCGR3B* gene could be used in adoptive cellular therapies to curb COVID-19-associated symptoms.

### Limitations of the study

The small sample size of this cohort is a limitation (31 patients with severe COVID-19 and 11 healthy controls). Although we did qPCR validation for *FCGR3B* using nasopharyngeal swabs (due to availability), the ideal validation sample would be flow cytometry-sorted macrophages from BALF region.

## STAR★Methods

### Key resources table

**Table:** 

REAGENT or RESOURCE	SOURCE	IDENTIFIER
**Bacterial and Virus Strains**		

SARS-CoV-2 spike protein (S1+S2)	Sino Biological Inc	Cat# 40591-V08H, 40590-V08B

**Biological Samples**		

Nasopharyngeal swab • Covid19 severe patients • Control	Dubai Health Authority	DSREC-04/2020_02

**Chemicals, Peptides, and Recombinant Proteins**		

RiboZol RNA extraction reagent	VWR	Cat# DFU-N580

**Critical Commercial Assays**		

QIAamp Viral RNA Mini or the EZ1 DSP Virus Kits	Qiagen	Cat# 955134
RNeasy 96 QIAcube HT Kit	Qiagen	Cat# 74171
High-Capacity cDNA Kit	Applied Biosystems	Cat# 4368814
Taqman Fast advanced master mix	ThermoFisher Scientific	Cat# 4444557
AccuRT Genomic DNA Removal Kit	Applied Biological Materials	Cat# G488
All-In-One Reverse Transcriptase Mastermix	Applied Biological Materials	Cat# G592
EvaGreen qPCR Mastermix	Applied Biological Materials	Cat# Mastermix-S

**Deposited Data**		

Healthy control airway scRNAseq	Deprez et al., 2020	https://www.genomique.eu/cellbrowser/HCA/HCA_airway_epithelium/exprMatrix.tsv.gz
Healthy control lung scRNAseq	Madissoon et al., 2019	https://cellgeni.cog.sanger.ac.uk/tissue-stability/lung.cellxgene.h5ad
COVID-19 Patient BALF scRNAseq	Liao et al., 2020	GEO: GSE145926
COVID-19 Patient bulk PBMC RNAseq	Arunachalam et al., 2020	GSE152418
COVID-19 Patient bulk nasopharyngeal RNAseq		GSE152075
COVID-19 Patient BALF scRNAseq	Grant et al., 2021	GSE155249

**Experimental Models: Cell Lines**		

Normal human primary bronchial epithelial (NHBE) cells from non-obese and obese subjects	MatTek and ATCC or obtained from the Biobank of the Quebec Respiratory Health Research Network at the Meakins-Christie Laboratories, Research Institute of the McGill University Health Center (GLEN site)	NA

**Oligonucleotides**		

FCGR3B qPCR probe	Applied Biosystems	Hs04334165_m1
FFAR2 qPCR probe	Applied Biosystems	Hs00271142_s1
glyceraldehyde-3-phosphate dehydrogenase qPCR probe	Applied Biosystems	Hs02786624_g1

**Software and Algorithms**		

Scanpy (version:1.7.0)		https://scanpy.readthedocs.io/en/stable/
Seurat (version: 3.2)		https://satijalab.org/seurat/
Custom Source Code	Github Folder: MBRULab/2021_Nassir-etal	https://github.com/MBRULab/2021_Nassir-etal

**Other**		

PanglaoDB	Franzen et al., 2019	https://panglaodb.se/
CellMarker	Zhang et al., 2019	http://biocc.hrbmu.edu.cn/CellMarker/
Immunology Database and Analysis Portal	Bhattacharya et al., 2018	https://www.immport.org/
Cytoscape		https://cytoscape.org/
Sfari		https://gene.sfari.org/
GWAS catalog		https://www.ebi.ac.uk/gwas/

### Resource availability

#### Lead contact

Further information and requests for resources and reagents should be directed to and will be fulfilled by the corresponding author, Mohammed Uddin (mohammed.uddin@mbru.ac.ae).

#### Materials availability

This study did not generate new unique reagents.

### Experimental model and subject details

#### Human subjects and ethics approval

Sociodemographic and clinical data were extracted from the electronic medical records of the patients with laboratory confirmed SARS-CoV-2 from 20 April to 06 May 2020 using the WHO case report form. Cases were categorized into two groups based on disease severity: severe/critical cases with advanced disease and pneumonia requiring admission to intensive care units and specialized life-support treatment (e.g., mechanical ventilation) and control (SARS-CoV-2 negative). This study was approved by the Dubai Scientific Research Ethics Committee-Dubai Health Authority (approval number #DSREC-04/2020_02). The requirement for informed consent was waived as this study was part of a public health surveillance and outbreak investigation in the UAE. Nonetheless, all patients treated at a healthcare facility in the UAE provide written consent for their deidentified data to be used for research and this study was performed in accordance with the relevant laws and regulations that govern research in the UAE. The age and sex data for the cohort is included in Table S5.

### Method details

#### Single-cell transcriptome datasets and clustering

The single-cell RNA-seq data for control and COVID-19 patients were obtained from three different studies. The first control dataset was assembled using the expression matrix and the metadata for the human healthy airway downloaded from Deprez et al. (2020). This study involved 35 samples collected from 4 distinct locations of 10 healthy volunteers: the nose (lower turbinate), the trachea/carina, intermediate bronchi, and distal bronchi. The sampling methods used were (superficial/luminal) brushings and (deep) biopsies. 77,969 cells were collected in total (having the largest cell size present within the nasal region) with 1,892 expressed genes detected per cell. The second control dataset was retrieved using the healthy lung’s H5AD file consisting of 57,020 cells from 4 donors (Madissoon et al., 2019). In this case, spleen, esophagus, and lung samples were collected from deceased donors and placed in HypoThermosol FRS solution for 12 to 72 hours (stored at 4°C), and the quality of collected samples was monitored at regular time intervals. The third dataset was retrieved from bronchoalveolar lavage fluid (BALF) single cell (Liao et al., 2020). BALF was collected from 3 healthy controls (control dataset 3) and 9 hospitalized COVID-19 patients, 3 of which had a moderate infection (Moderate COVID-19 dataset) and the rest had a severe disease course (Severe COVID-19 dataset).

The clusters for all the datasets were reproduced mostly using the standard analysis steps (Deprez et al., 2020; Liao et al., 2020; Madissoon et al., 2019; Tambi et al., 2021) as mentioned in their respective research articles to recapitulate the basic cluster topology. Scanpy (version:1.7.0) was used for processing the control dataset 1 and 2. Single-cell object was constructed from the expression matrix and the metadata downloaded for the control dataset1. PCAs were computed and the highly variable 12 PCs were selected from the elbow plot for calculating the neighbors. Furthermore, PhenoGraph which employs unsupervised k-nearest neighbor (KNN) technique was used to detect the clusters and the cluster connectivities were calculated using the partition-based graph abstraction (PAGA) method which is an additional step used in our analysis (not used by Deprez et al.) to help precisely mark the clusters of large single-cell datasets. Finally, UMAP was used for visualizing the clusters in a reduced dimensional space and the reproduced clusters were topologically compared with the UMAP from Deprez *et al.* The availability of H5AD object for the control dataset 2, facilitated the replication of the clusters. In case of the BALF dataset (Liao *et al*), we processed the healthy control, moderate and severe samples separately. The batch corrected single cell transcriptome matrix (h5seurat object) was used for downstream analysis in our study. Batch correction includes canonical correlation analysis (CCA) to find the correlations across datasets, following which the mutual nearest neighbors aiding as ‘anchors’ are computed in the CCA subspace to correct the data. We used Seurat (version: 3.2) to analyze 21939, 7316, 37197 cells for the control, moderate and severe samples, respectively. The cells were filtered based on quality control metrics which involved filtering the cells which had less than 200 feature counts and features expressed in less than 3 cells. The filtered data were normalized (log(expression/total∗10000)) using the ‘LogNormalize’ function, the data were scaled, principal components were calculated and using the top PCs showing maximum variance, clusters were computed using ‘FindClusters’ which were visualized using UMAP. Different single cell transcriptome data were analyzed separately. Hence, each data do not impact the other and downstream analyses has negligible batch effect after the standard corrections. Eventually, the differentially expressed genes (DEG) were identified for each computed cluster using three statistical tests: Wilcoxon-ranked sum test, t-test, t-test overestimated variance (Table S9). DEGs were considered if all three tests showed significance. DEGs were computed using the ‘FindMarker’ function of Seurat and the probability value were estimated with respect to all other clusters within each dataset.

#### Cluster identity analysis

An in-house database consisting of canonical markers for cells associated to human lung region was constructed using a combination of literature search (Deprez et al., 2020; Grant et al., 2021; Madissoon et al., 2019; Reyfman et al., 2019), PanglaoDB (Franzen et al., 2019), and CellMarker (Zhang et al., 2019) databases. This dataset was curated by merging the recurring cell type and duplicate genes were removed. Our final marker database consisted of 38 cell types represented by a total of 966 unique marker genes (Table S1). The cell type specific genes were mapped onto the clusters. Box plots were used to analyze their expression and the cell type identity was assigned based on the highest normalized median expression value for each cluster (Table S2).

#### COVID-19 comorbid genes

A list of COVID-19 associated genes (Table S3) was collated to analyze and compare their expression among control, moderate and severe COVID-19 clusters. Since COVID-19 is supplemented by an enormous release of pro-inflammatory cytokines, the list consisted of cytokine and cytokine receptor genes, in addition to the rare infection and syndromic genes accounting for the genetic predisposition, lung channel genes, differentially expressed genes from COVID-19’s BALF transcriptome (Zhou et al., 2020b) and genes associated with key comorbid conditions such as chronic obstructive pulmonary disorder, cardiovascular disease, hypertension and diabetes. The list of cytokine and cytokine-receptor genes were retrieved from the immunology Database and Analysis Portal (Bhattacharya et al., 2018). The monogenic infection gene list was retrieved (Casanova, 2015). Syndromic genes (category S) were collected from the Simons Foundation Autism Research Initiative (https://gene.sfari.org/). Primary comorbid disease genes associated with Covid-19 were extracted from GWAS catalog (https://www.ebi.ac.uk/gwas/), where the selected genes had a probability < 10-7. The single cell dataset was annotated for these 10 gene lists. The normalized expression of these genes was plotted for all the clusters of the control, moderate and severe datasets.

#### Pathway analysis

Gene enrichment analysis using R (Gene Overlap package) and Cytoscape was used to analyze the pathways specifically for severe cluster 11. The enriched set of genes along with their gene set (GMT) file (consisting of KEGG and GO pathways) were uploaded in the Cytoscape. The minimum number of gene overlap was set to 5 and the false decision rate and p-value cut off was 0.01 and 0.001, respectively. Then, the network was built using the enrichment map and the auto annotate plugin in Cytoscape application. The node color (red-white gradient) and size (low to high) was set based on increasing p-value and odd’s ratio, respectively.

#### Gene expression dataset for validation

We selected the top 20 genes upregulated in cluster 11 based on their fold change values and further segregated the cluster 11 restrictive marker genes by examining their expression patterns in feature plots, dot plots and box plots. Three RNAseq dataset were used to analyze the expression of the cluster specific marker genes. Two bulk transcriptomic dataset (GSE152418 and GSE152075) and one single cell data (GSE155249) were downloaded from the NCBI Gene Expression Omnibus (GEO) database. GSE152418 consisted of peripheral blood mononuclear cells (PBMC) RNAseq data from 17 COVID-19 and 17 healthy subjects. The expression matrix consisted of raw counts for 60,683 genes. GSE152075 consisted of nasopharyngeal RNAseq data from 430 COVID-19 subjects and 54 negative controls. The expression matrix consisted of raw counts for 35,785 genes. We used ‘cpm’ from edgeR package to generate counts per million values. It was used to generate the ‘DGEList’ object and we finally calculated the ‘log’ of the cpm values which were used in further analysis. Further, we used BALF single cell data GSE155249 to analyze the marker gene expression. This dataset consisted of samples from five severe COVID-19 patients and one control from a patient with bacterial pneumonia. The expression matrix consisted of 11,217 and 28,114 cells of the control and COVID-19 samples, respectively, for 20,128 genes. The expression data was normalized using “LogNormalize” method which divides the individual gene expression for a cell by the total expression, multiplies it by a scale factor of 10,000 (default), and log-transforms the product. The gene expression between the control and COVID-19 samples were compared and plotted using the top 5% data.

#### Extraction of total human RNA from nasopharyngeal swabs

The nasopharyngeal swabs of severe hospitalized and in ICU COVID-19 patients tested positive (n=31) for SARS-CoV-2 by RT-qPCR following the QIAamp Viral RNA Mini or the EZ1 DSP Virus Kits (Qiagen, Hilden, Germany) and the control (qPCR negative) swabs (n=11) were used in the study. Human RNA was extracted from nasopharyngeal swabs using RNeasy 96 QIAcube HT Kit (Qiagen USA) as per the manufacturer’s instructions. 2 ml of Universal Transfer Medium (UTM) was transferred to a clean, sterile Eppendorf tube and centrifuged at 4000 rpm for 3 min. 1650 μl of supernatant was carefully removed without disturbing the pelleted squamous and respiratory epithelial cells. Pellet was resuspended with the remaining 350 μl of Universal Transfer Medium (UTM) and transferred to S Block (RNeasy 96 QIAcube HT Kit, Qiagen USA). Quality and quantity of the extracted RNA was determined using NanoDrop™ 8000 Spectrophotometer (ThermoFisher Scientific, USA) and reverse transcribed to cDNA using a High-Capacity cDNA Kit (Applied Biosystems, Foster City, CA) according to the manufacturer's protocol.

#### qPCR of *FCGR3B* and *FFAR2* in severe COVID-19 patients

Quantitative real-time RT-PCR was performed using the Quantstudio 5 Real-time PCR detector with the following probes from Applied Biosystems (Foster City, CA): *FCGR3B* (Hs04334165_m1), *FFAR2* (Hs00271142_s1), and glyceraldehyde-3-phosphate dehydrogenase (Hs02786624_g1) using Taqman Fast advanced master mix (ThermoFisher Scientific, USA). Hs04334165_m1 design was chosen avoiding homology of other FCR gene family transcripts. Samples were prepared in a 96-well plate format in triplicates along with no template control. The cycling conditions consisted of an initial incubation at 50°C for 2 min, polymerase activation at 95°C for 5 min, followed by 40 amplification cycles at 95°C for 1 s, 60°C for 20 s. Relative levels of mRNA gene expression were calculated using the 2−ΔΔCT method and fold change was plotted.

#### Lung epithelial cell culture

Normal human primary bronchial epithelial (NHBE) cells from non-obese and obese subjects were purchased from a commercial source (MatTek, MA, USA and ATCC, VA, USA) or obtained from the Biobank of the Quebec Respiratory Health Research Network at the Meakins-Christie Laboratories, Research Institute of the McGill University Health Centre (GLEN site). Table S6 shows the data from the non-obese and obese subjects. NHBE cells were cultured in BEGM media (Lonza, MD, USA) supplemented with 1% antibiotic antimycotic solution (Wisent, QC, CA) in tissue culture 12 well plates coated with Type 1 Rat tail collagen (Sigma-Aldrich, Ontario, Canada). Cells were grown to 90% confluency and starved using BEBM Basal Medium (Lonza, MD, USA) supplemented with 1% antibiotic antimycotic solution (Wisent, QC, CA) over night. The next day cells were stimulated with 1ug/ml of SARS-CoV-2 spike protein (S1+S2) (Sino Biological Inc., Beijing, China) for 3h. Culture media was collected in microtubes, centrifuged 5000g for 5 min. Supernatants were aliquoted into fresh microtubes and frozen at -80°C.

#### Monocyte isolation and culture

PBMCs from a healthy donor were isolated from 40ml of blood using SepMate-50 tubes (StemCell, BC, CA), following manufactures protocol. PBMCs were re-suspended in RPMI 1640 supplemented with 1% Penicillin/Streptomycin, Glutamax and 10% FBS (Wisent, QC, CA) (Gibco, MD, USA) (Wisent, QC, CA). A differential cell count was performed on the PBMC cell suspension using the Beckman Coulter AC-T DIFF cell counter and monocytes concentration was obtained. Calculated volume of PBMCs cell suspension was added to a 48 well plate to obtain 1x10ˆ5 monocytes per well. Cell suspension was incubated at 37°C, 5% CO2 for 3 h. Media from wells was removed and washed twice with PBS. Fresh complete RPMI media with 0.5% FBS was added to each well and attached monocytes were incubated overnight. The following day an aliquot of conditioned media from stimulated NHBE cells was thawed on ice and vortexed. 100ul of media was removed from wells and 100ul of NHBE conditioned media was added to attached monocytes and incubated for 24 h. Media was then removed from cells and cell pellets were used for RNA extraction.

#### RNA extraction and quantitative reverse transcription polymerase chain reaction (qPCR)

Extraction of total RNA from monocytes was preformed using phenol-chloroform extraction (RiboZol RNA extraction reagent, VWR, Leicestershire, UK), as directed in the manufacturer’s instructions. Contaminated DNA was removed from 500 ng of total RNA using the AccuRT Genomic DNA Removal Kit (Applied Biological Materials, Richmond, BC, Canada), following the manufacturer’s protocol. Reverse transcription was preformed using the 5X All-In-One Reverse Transcriptase Mastermix (ABM). mRNA expression of *FCGR3B* and *GAPDH* (housekeeping gene) were measured using EvaGreen qPCR Mastermix (ABM). Table S7 shows forward and reverse primers used. The reaction was as follows: 5 μl of EvaGreen Mastermix, 2 μl of diluted cDNA (1/5), 0.6 μl of forward and reverse primers (10 μM) and 2.4 μl of nuclease free H2O. Each sample was tested in duplicates and the qPCR amplification was performed using CFX96 thermal cycler (BioRad, Hercules, 130 CA, USA) and cycler conditions were set according to the manufacturer’s protocol. The ΔCT method was used to measure gene expression: amount of target = CT ref /ΔCT.

### Quantification and statistical analysis

Differential gene expression was analyzed using three tests, Wilcoxon-ranked sum test, t-test and t-test overestimated variance. DEGs were computed using the ‘FindMarker’ function of Seurat and the probability value were estimated with respect to all other clusters within each dataset. Pathway enrichment was analyzed using GSEA method in the “fgsea” package. Statistical significance of normalized enrichment scores was qualified, with a significance threshold of false decision rate and p-value cut off at 0.01 and 0.001, respectively. To quantify statistical significance, we have used R package and applied t-test and Fisher’s exact test (qPCR).

## Data Availability

Multi-donor datasets from three separate studies were used in this work and these can be found under the following accession numbers: GEO: GSE145926 (Liao et al., 2020), https://www.genomique.eu/cellbrowser/HCA/HCA_airway_epithelium/exprMatrix.tsv.gz (Deprez et al., 2020), https://cellgeni.cog.sanger.ac.uk/tissue-stability/lung.cellxgene.h5ad (Madissoon et al., 2019). Additional Supplemental Items are available from Mendeley Data at https://data.mendeley.com/datasets/mkz6jfywmd/1, https://doi.org/10.17632/4fg7c8t568.1. Analyses were conducted in R and Python; all codes for analyses have been deposited at Github and is available online at https://github.com/MBRULab/2021_Nassir-etal. Any additional information required to reanalyze the data reported in this paper is available through personnel contact with the corresponding author upon request.

## Supplemental information

Documents S1. Figures S1–S16 and Tables S6, S7 and S10–S12

Table S1. Cell type marker list, related to Figure 2

Table S2. Cell identity of severe, moderate and control clusters, related to Figure 2

Table S3. List of comorbid genes, related to Figure 2

Table S4. Pathway enrichment of Cluster 11, related to Figure 2

Table S5. Real-time PCR of FCGR3B in clinical samples, related to Figure 4

Table S8. Real-time PCR of FCGR3B in monocyte experiment, related to Figure 4

Table S9. DE genes of severe clusters, related to Figure 2
