# Supplementary material for: Single-cell transcriptome identifies FCGR3B upregulated subtype of alveolar macrophages in patients with critical COVID-19
Source: iScience. 2021 Aug 25;24(9):103030. doi: 10.1016/j.isci.2021.103030 (PMC8384759; doi:10.1016/j.isci.2021.103030)
Supplement: Documents S1. Figures S1–S16 and Tables S6, S7 and S10–S12 [file mmc1.pdf]

iScience, Volume *24*

Supplemental information

Single-cell transcriptome identifies *FCGR3B* upregulated subtype of alveolar macrophages in patients with critical COVID-19

Nasna Nassir, Richa Tambi, Asma Bankapur, Saba Al Heialy, Noushad Karuvantevida, Hamda Hassan Khansaheb, Binte Zehra, Ghausia Begum, Reem Abdel Hameid, Awab Ahmed, Zulfa Deesi, Abdulmajeed Alkhajeh, K.M. Furkan Uddin, Hosneara Akter, Seyed Ali Safizadeh Shabestari, Omar Almidani, Amirul Islam, Mellissa Gaudet, Richard Kumaran Kandasamy, Tom Loney, Ahmad Abou Tayoun, Norbert Nowotny, Marc Woodbury-Smith, Proton Rahman, Wolfgang M. Kuebler, Mahmood Yaseen Hachim, Jean-Laurent Casanova, Bakhrom K. Berdiev, Alawi Alsheikh-Ali, and Mohammed Uddin

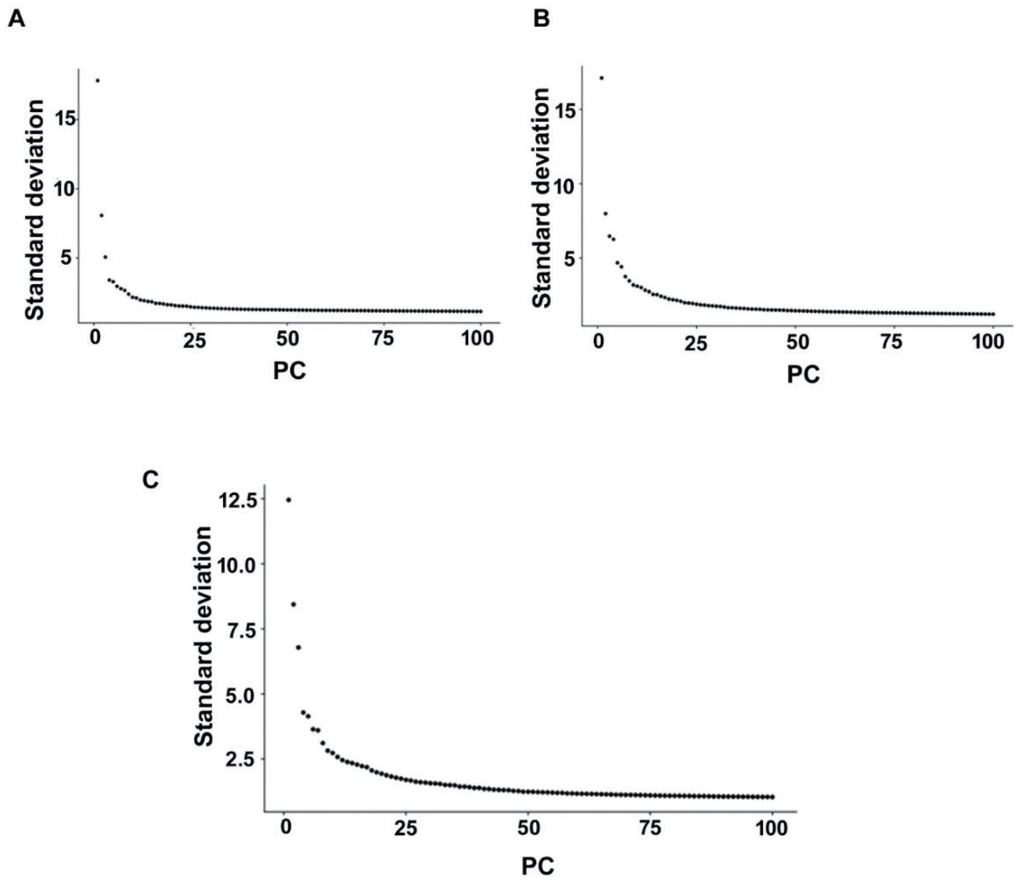

Figure S1. Seurat generated elbow plot to determine the number of principal components. Related to Figure 1 A) Control. B) Moderate and C) Severe BALF dataset. The number of PCs is represented on the x-axis and their standard deviation on y-axis. The control, moderate and severe BALF consisted of 21939, 7316, 37197 cells, respectively. The cells were filtered based on quality control metrics and the filtered data was normalized and scaled after which the dimensionality reduction was performed using 'RunPCA' using the most variable features. We selected 11 PCs for all the three dataset and calculated the clusters which were visualized using UMAP.

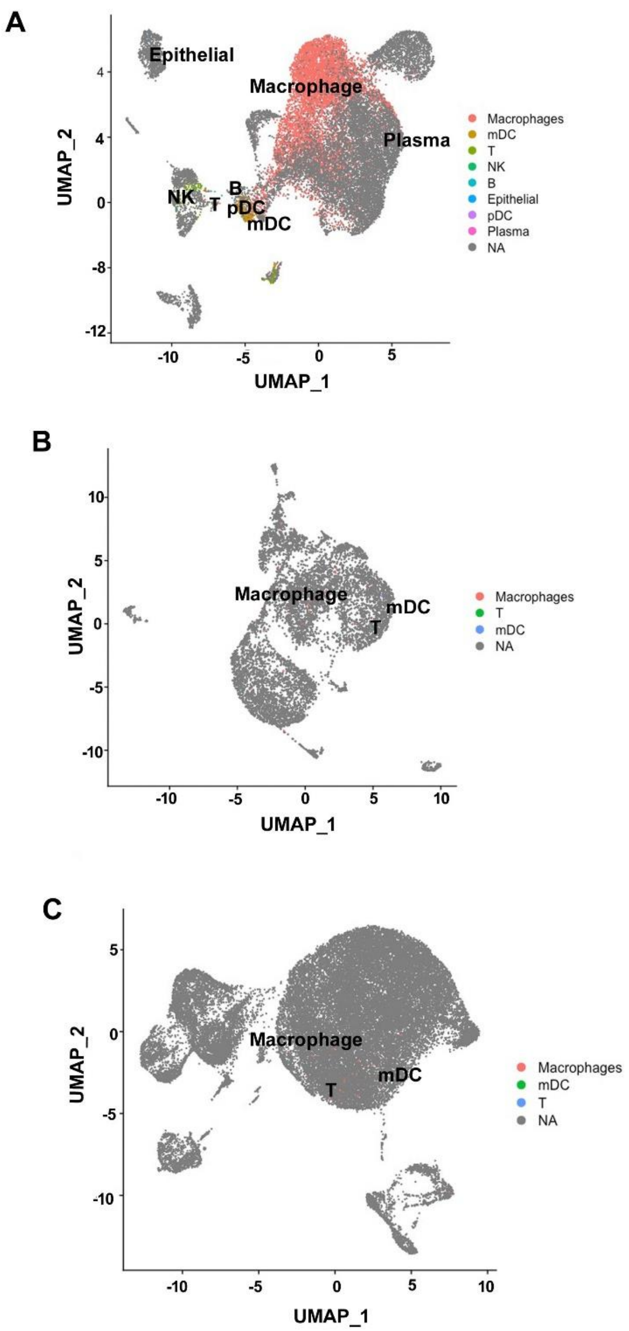

Figure S2. Mapping the transcriptomic dataset identity. Related to Figure 1. A) Control. B) Moderate. C) Severe BALF clusters using Liao et al markers. We segregated the Liao et al. single cell BALF data, into control, moderate, and severe samples and generated individual single cell maps for each group using standard Seurat protocol. The initial BALF data consisted of 31 clusters comprising of Neutrophil, Epithelial, Plasma, Mast, T-cell, B-cell, NK cell, pDC/mDC (plasmacytoid/myeloid dendritic cell) and Macrophages. However, when we used the same marker genes from Liao et al, we were not able to classify the cell identity for the segregated BALF data.

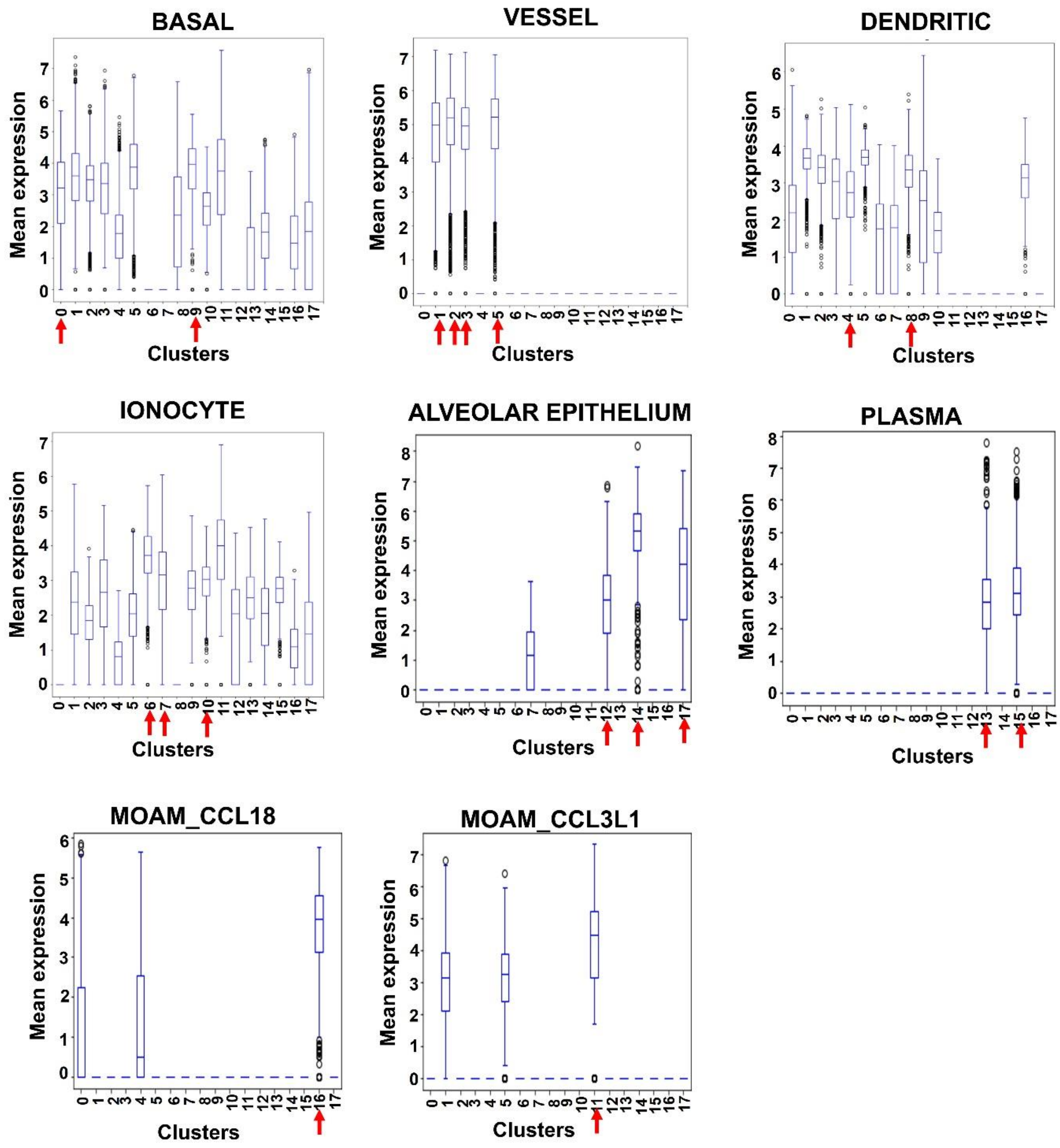

Figure S3. Boxplot showing the expression of signature marker genes for assigning cell type for the severe BALF clusters. Related to Figure 2. The mean expression (y-axis) was plotted for 17 severe BALF clusters (x-axis). 'Red arrow' placed below the cluster number indicates its corresponding cell identity. We used our in-house database which consisted of 966 unique gene markers for 38 different cell types associated to human lung region, to ascertain the identity of these clusters. We examined the median expression value for all the cell types per cluster. A cluster was assigned a particular cell type if it had the highest median expression. When a single cell type marker gene was highly expressed in two or more different Seurat defined clusters, the later were

marked as subtype (using roman numbers). Cluster 0 is Basal I, Clusters 1-3 are Vessels I-III (endothelial cells both from blood and lymph vessels), Cluster 4 is Dendritic I, Cluster 5 is vessel IV, Clusters 6-7 are Ionocytes I-II, Cluster 8 is Dendritic II, Cluster 9 is Basal, Cluster 10 is Ionocyte III, Cluster 11 is Moam_CCL3L1 (CCL3L1 specific monocyte derived alveolar macrophage), Cluster 12 is Alveolar epithelium I, Cluster 13 is Plasma I, Cluster 14 is Alveolar epithelium II, Cluster 15 is Plasma II, Cluster 16 is Moam_CCL3L18 and Cluster 17 is Alveolar epithelium III. Y-axis represent the mean of log normalized expression value for marker genes across the cell clusters.

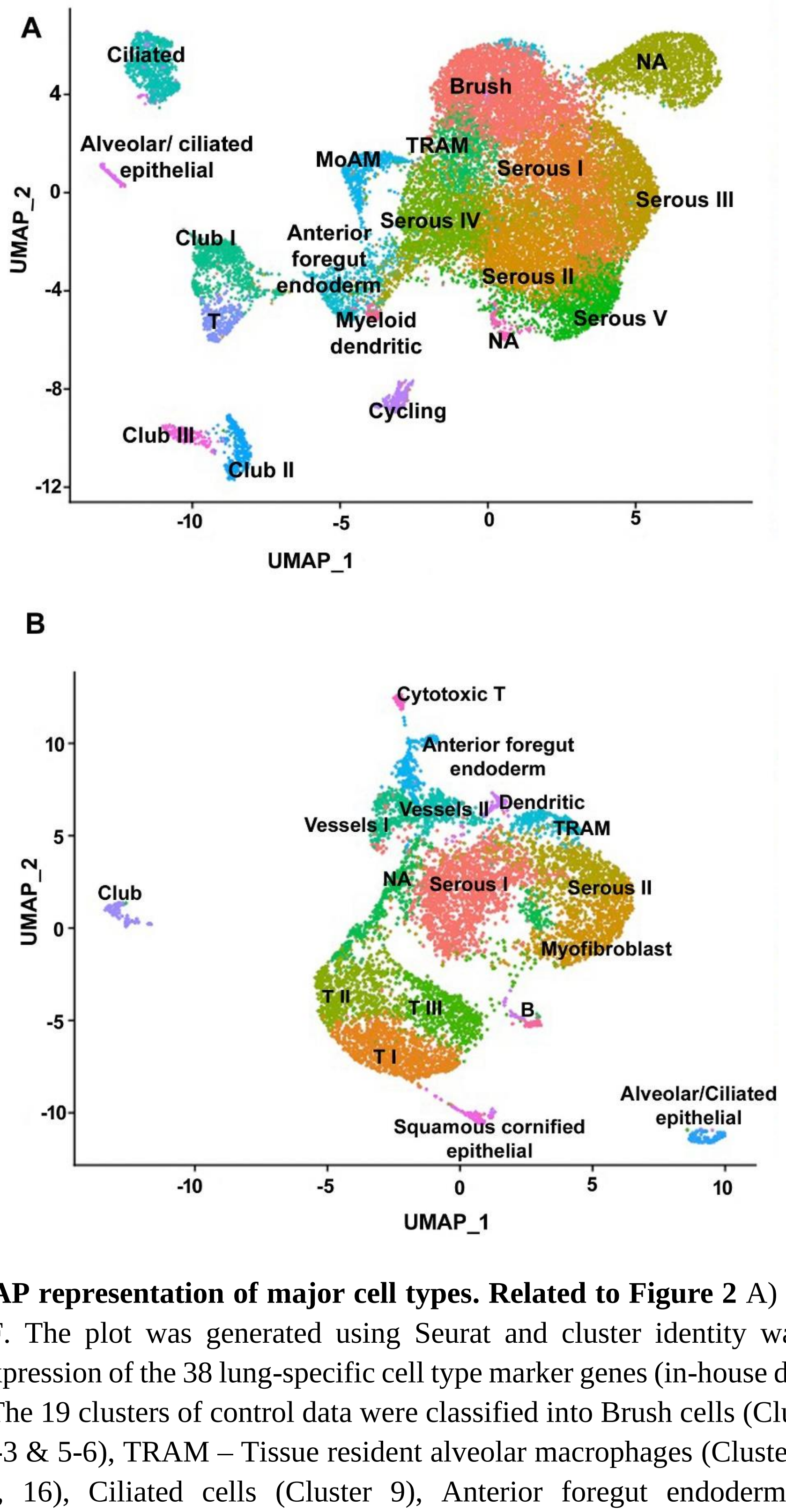

Figure S4. UMAP representation of major cell types. Related to Figure 2 A) Control and B) Moderate BALF. The plot was generated using Seurat and cluster identity was assigned by examining the expression of the 38 lung-specific cell type marker genes (in-house database) across all the clusters. The 19 clusters of control data were classified into Brush cells (Cluster 0), Serous cells (Clusters 1-3 & 5-6), TRAM – Tissue resident alveolar macrophages (Cluster 7), Club cells (Clusters 8, 12, 16), Ciliated cells (Cluster 9), Anterior foregut endoderm (Cluster 10),

Moam_CCL18 (Cluster 11), T cell (Cluster 13), Cycling cell (Cluster 14), Alveolar epithelium (Cluster 15) and mDC (Cluster 18). The 17 clusters of moderate consisted of Serous cells (Clusters 0 & 3), T cell (Clusters 1, 4 & 5), Myofibroblast (Cluster 2), Vessels (Clusters 7 & 8), TRAM (Cluster 9), Anterior foregut endoderm (Cluster 10), Alveolar epithelium (Cluster 11), Club cells (Cluster 12), Dendritic (Cluster 13). Squamous cornified epithelium (Cluster 14), Cytotoxic T-cell (Cluster 15) and B cells (Cluster 16). 'NA' stands for Not Assigned.

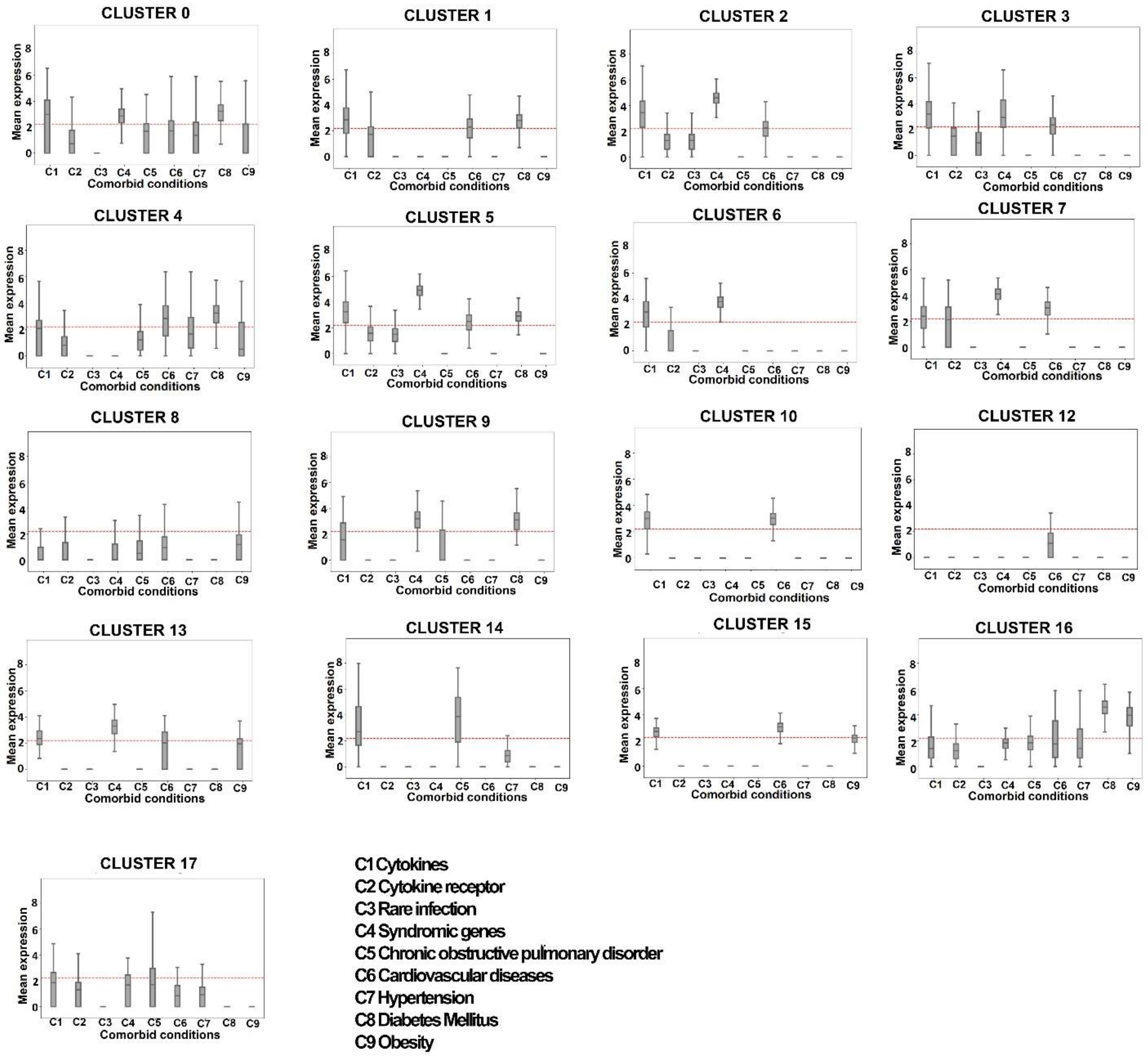

Figure S5. Boxplot showing the expression of comorbid condition associated genes across all the severe clusters except cluster 11. Related to Figure 2. The mean expression (y axis) of genes that are associated with nine comorbid diseases of severe COVID-19 (x axis) is shown in box plot. 99th percentile expression value (2.2) from the severe dataset is marked using red dotted lines. Y-axis represent the mean of log normalized expression value for comorbid genes across cell clusters.

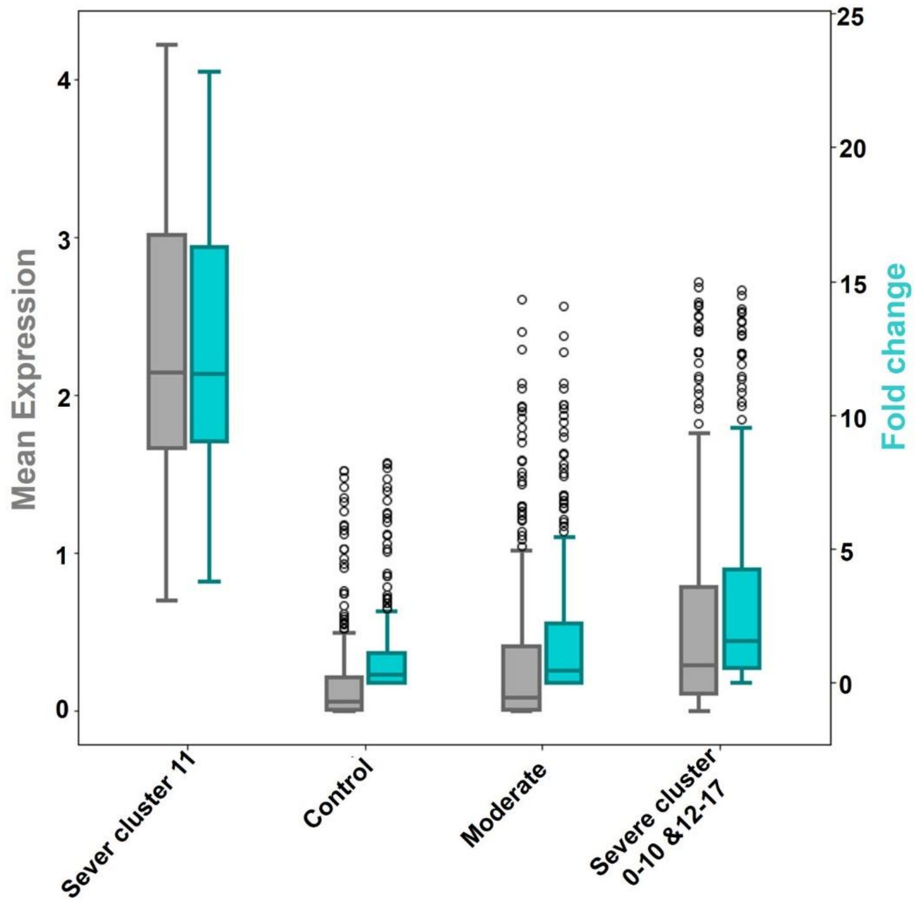

Figure S6. Comparison of the expression of top 20 genes in severe cluster 11 to control, moderate and all other severe clusters. Related to Figure 3. The top 20 genes were selected from severe cluster 11 based on fold change. This plot shows that the top 20 genes had higher mean expression (grey) and fold change (blue) in severe cluster 11 compared to control, moderate and other severe clusters. Y-axis represent the mean of log normalized expression value for top 20 genes across the cell clusters.

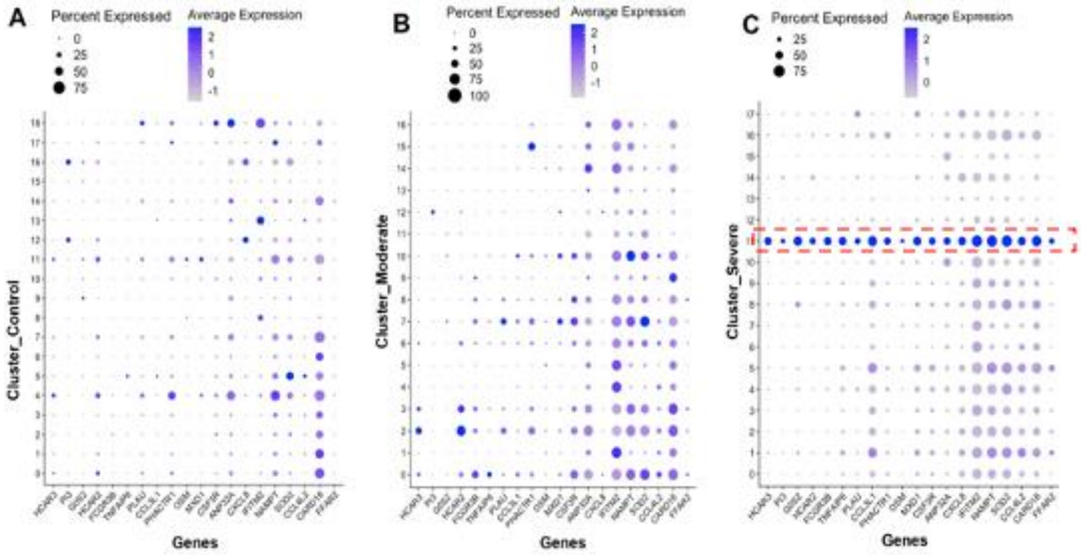

Figure S7. Dot plot showing the restrictive expression of top 20 genes of severe cluster 11. Related to Figure 3. A) Control. B) Moderate and C) Severe BALF. The gene names are on the x-axis and cluster numbers on the y-axis. Size of the circle correspond to the percent of cells per cluster in which the genes were expressed and colour gradient indicates the strength of expression. High restricted expression of genese within cluster 11 shown in red dotted box.

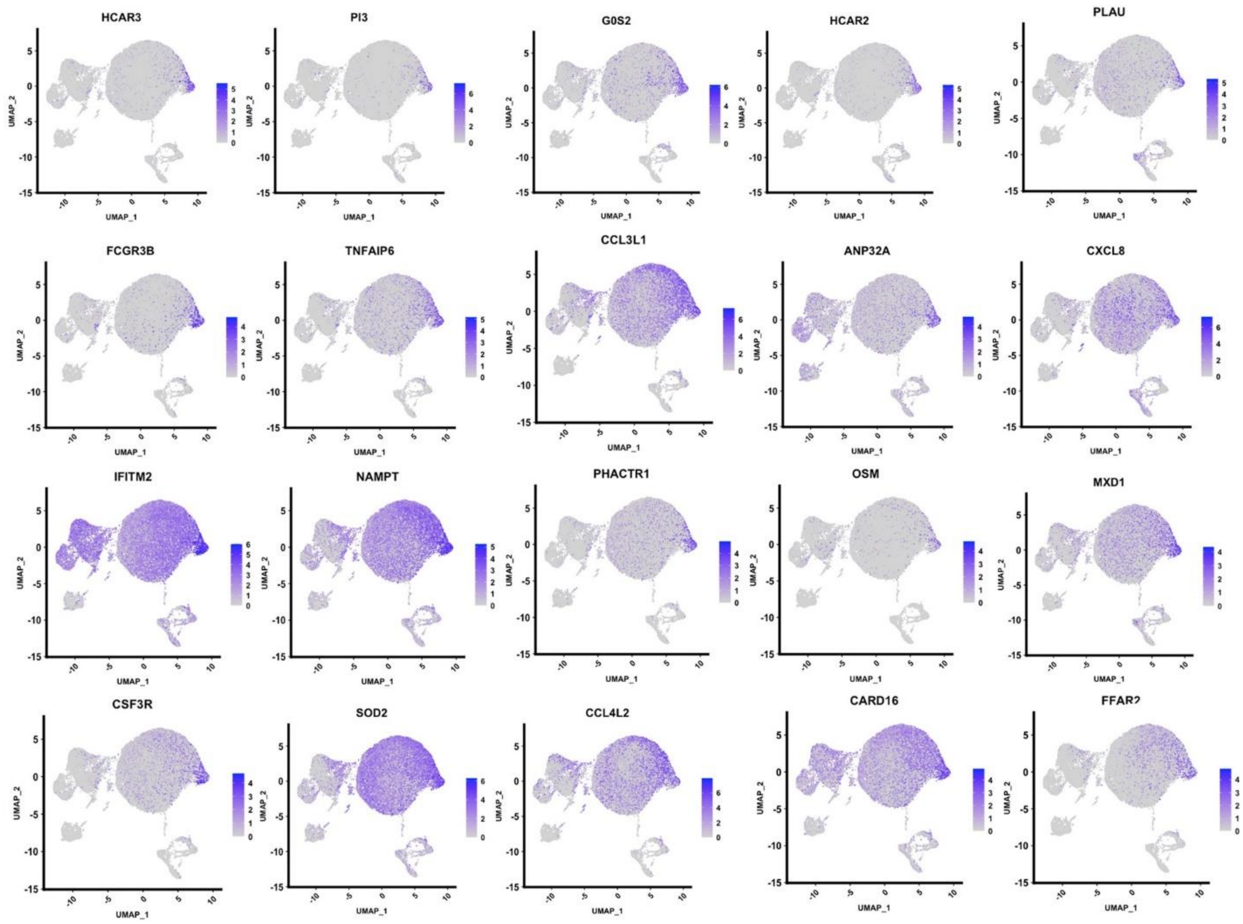

Figure S8. Feature plot depicting the expression of top 20 genes of severe cluster 11 across severe clusters. Related to Figure 3. The colour gradient represents the average expression of each gene as indicated in the legend.

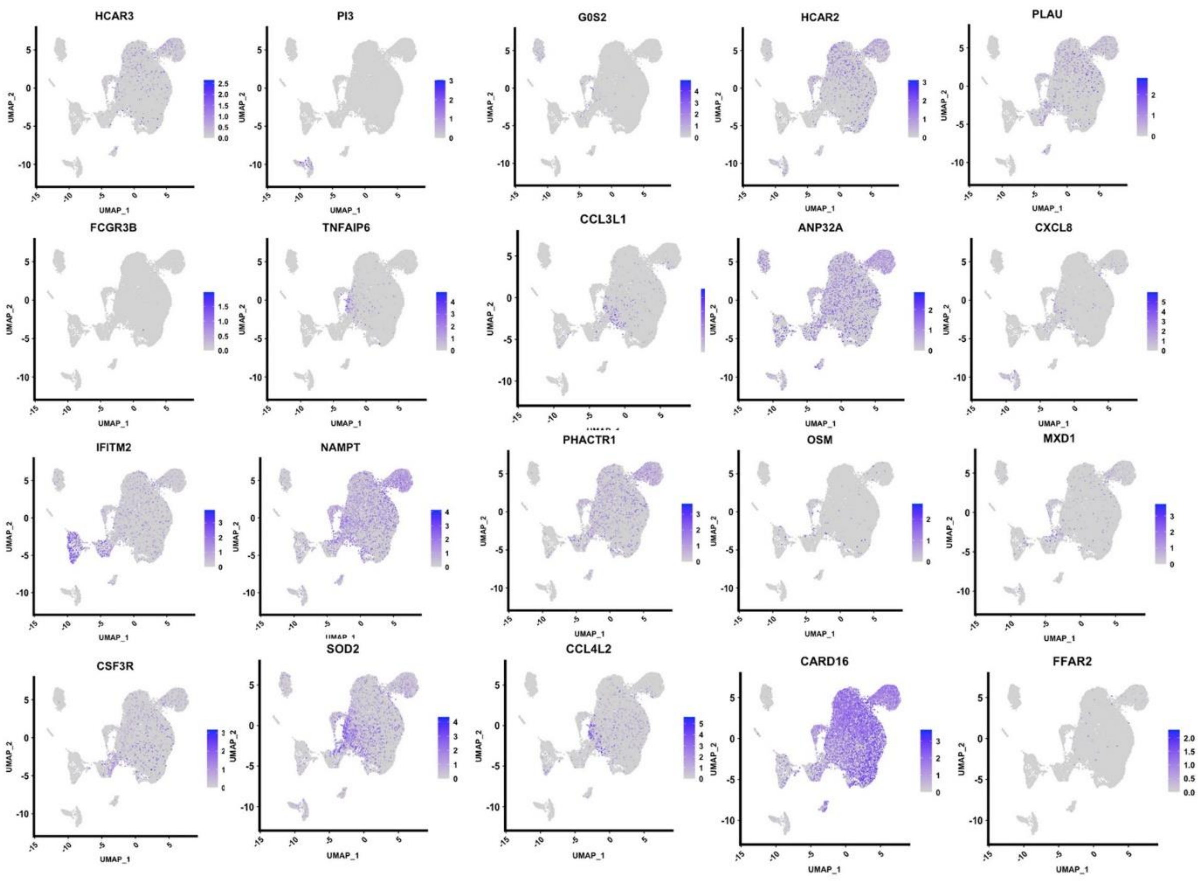

Figure S9. Feature plot depicting the expression of top 20 genes of severe cluster 11 across control clusters. Related to Figure 3. The colour gradient represents the average expression of each gene as indicated in the legend.

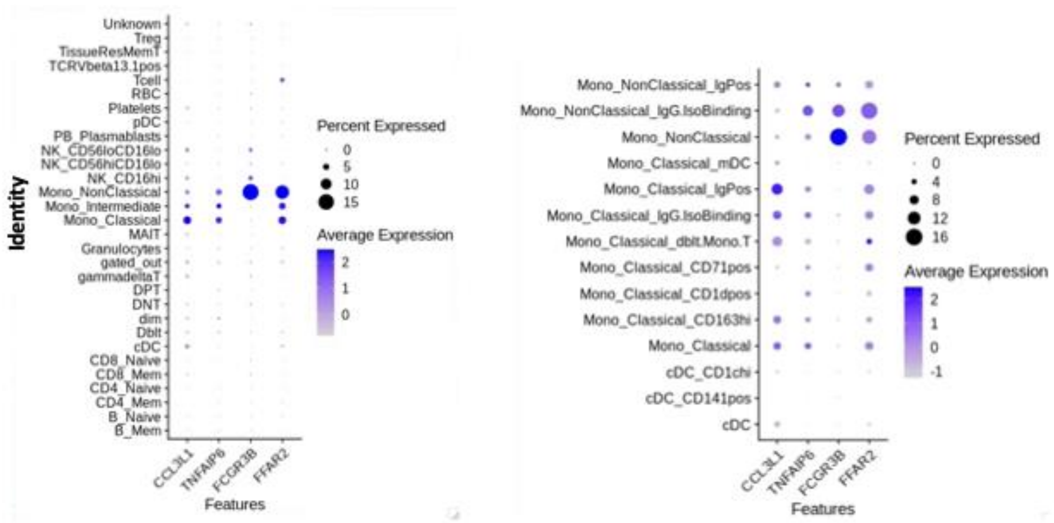

Figure S10. Dot plot depicting the expression of genes across different cell type. Related to Figure 3. Expression of CCL3L1, TNFA1P6, *FCGR3B* and *FFAR2* across immune cells, showing restrictive expression in non-classical monocytes (Liu C et al. 2021, Cell, in press, obtained through personnel communication). Size of the circle correspond to the percent of cells per cluster in which the genes were expressed and colour gradient indicates the strength of expression.

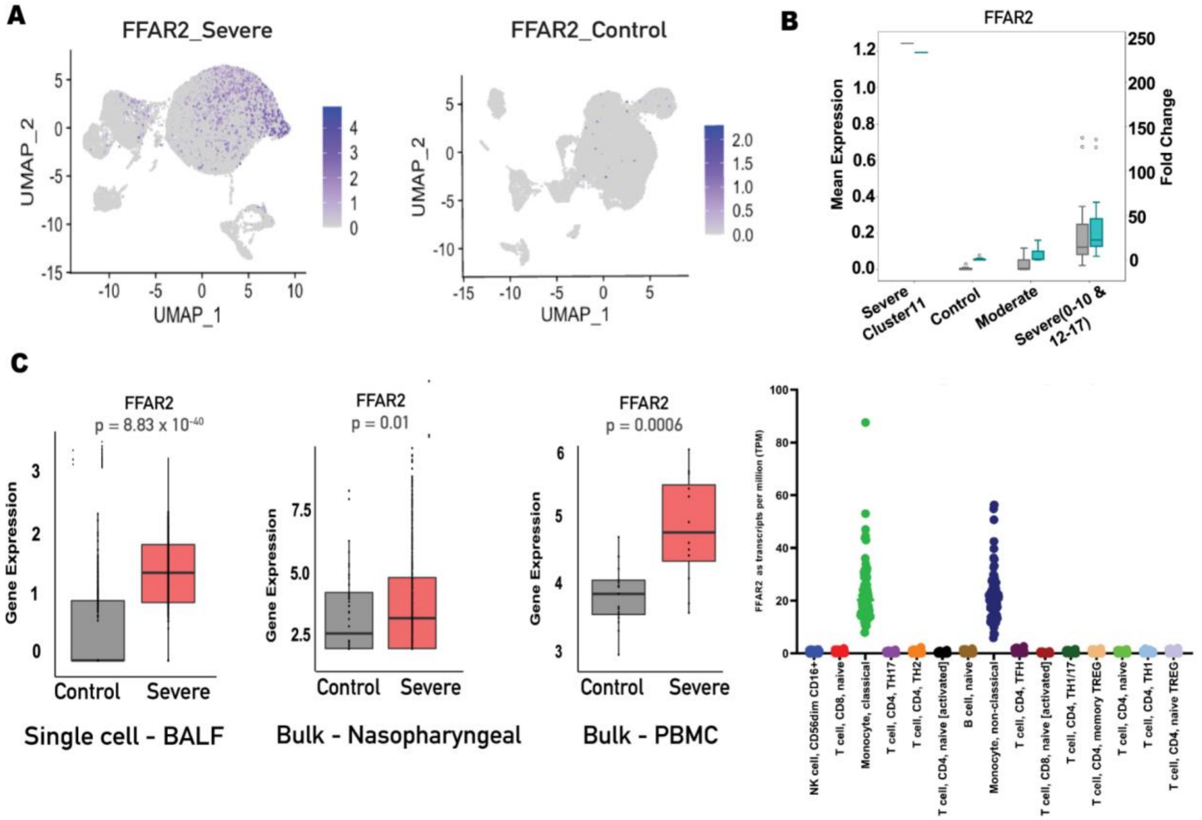

Figure S11. Cell type specific marker (*FFAR2*) characterization. Related to Figure 3. A) Feature plot showing restricted expression of *FFAR2* gene in severe cluster 11 and not in control. B) Boxplot showing higher mean expression (grey) and fold change (blue) of *FFAR2* gene in severe cluster 11 compared to control, moderate and other severe clusters. Y-axis represent the mean of log normalized expression value for each gene across the cells. C) Validation of expression of *FFAR2* gene across severe COVID-19 (red) and control samples (grey) in three independent databases (single cell BALF, bulk PBMC, bulk nasopharyngeal). In single cell BALF data, the y-axis represents the log normalized expression value calculated using Seurat. Expression counts per cell were divided by the total counts for that cell (sequencing depth) and multiplied by the 10000 (scale factor), which was then log transformed. For the bulk RNA seq data, y-axis represents the log transformed count per million expression value computed using EdgeR package. D) Dotplot showing higher restricted expression of *FFAR2* in non-classical monocytes of blood single cell transcriptome.

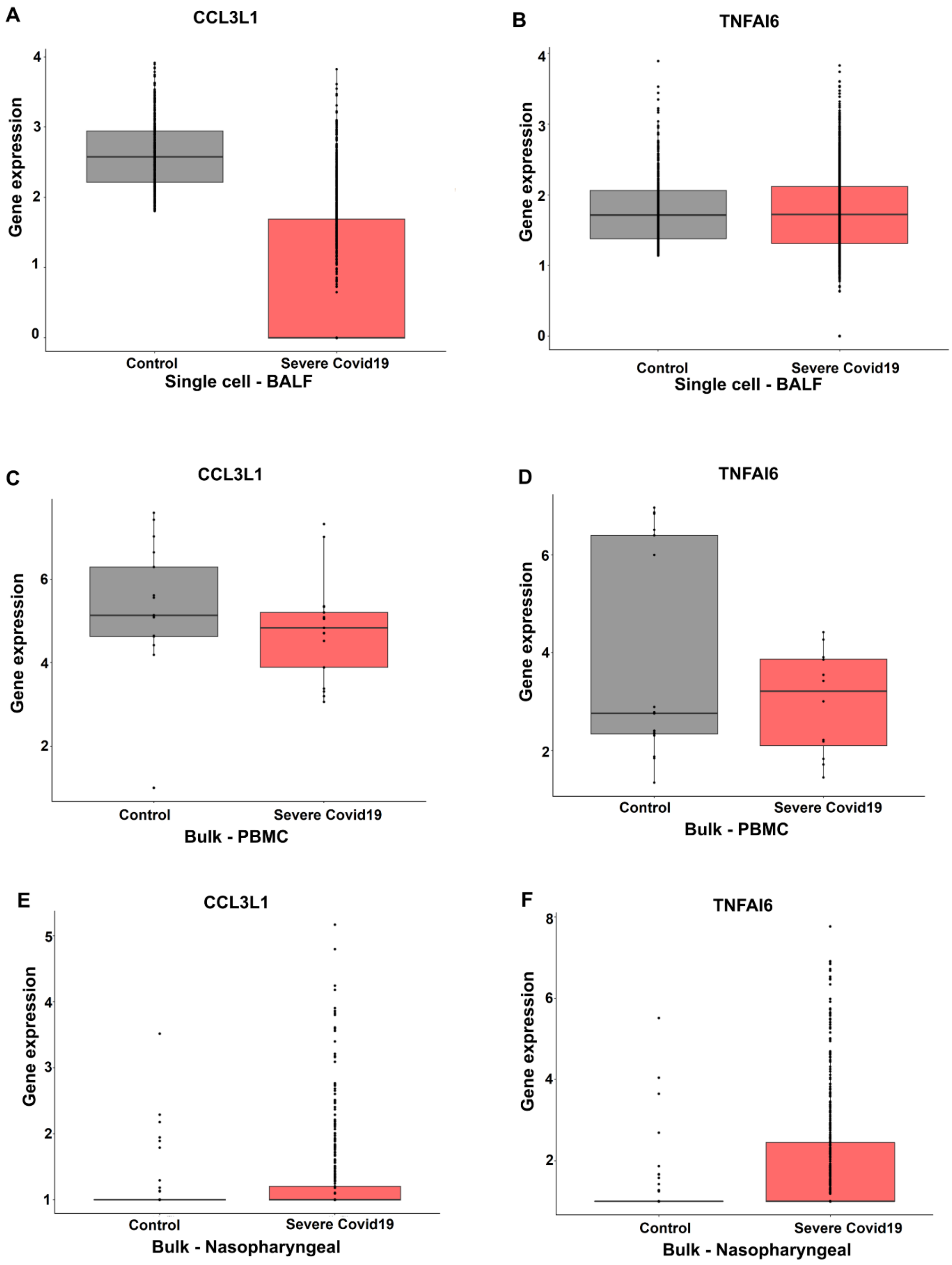

Figure S12. Expression of *CCL3L1* and *TNFAI6* across severe COVID-19 (red) and control samples (grey) in three independent databases (single cell BALF, bulk PBMC, bulk nasopharyngeal). Related to Figure 4. In single cell BALF data, the Y-axis represents the log normalized expression value calculated using Seurat. Expression counts per cell were divided by

the total counts for that cell (sequencing depth) and multiplied by the 10000 (scale factor), which was then log transformed. For the bulk RNA seq data, Y-axis represents the log transformed count per million expression value computed using EdgeR package.

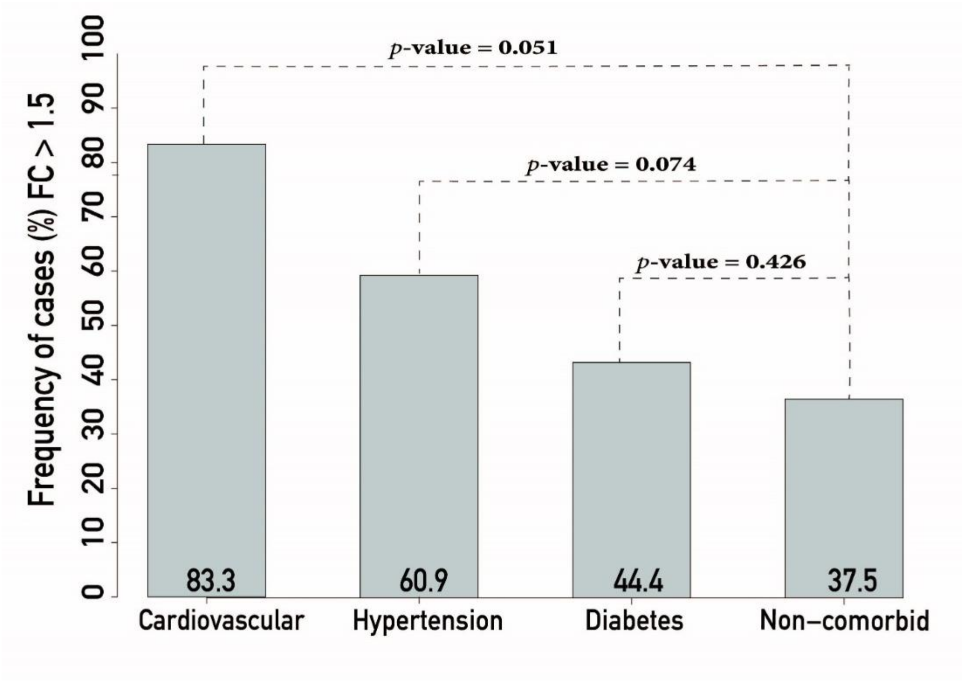

Figure S13. Frequency of cases with fold change greater than 1.5 of *FCGR3B* in different comorbid conditions of severe COVID-19 cases. Related to Figure 4.

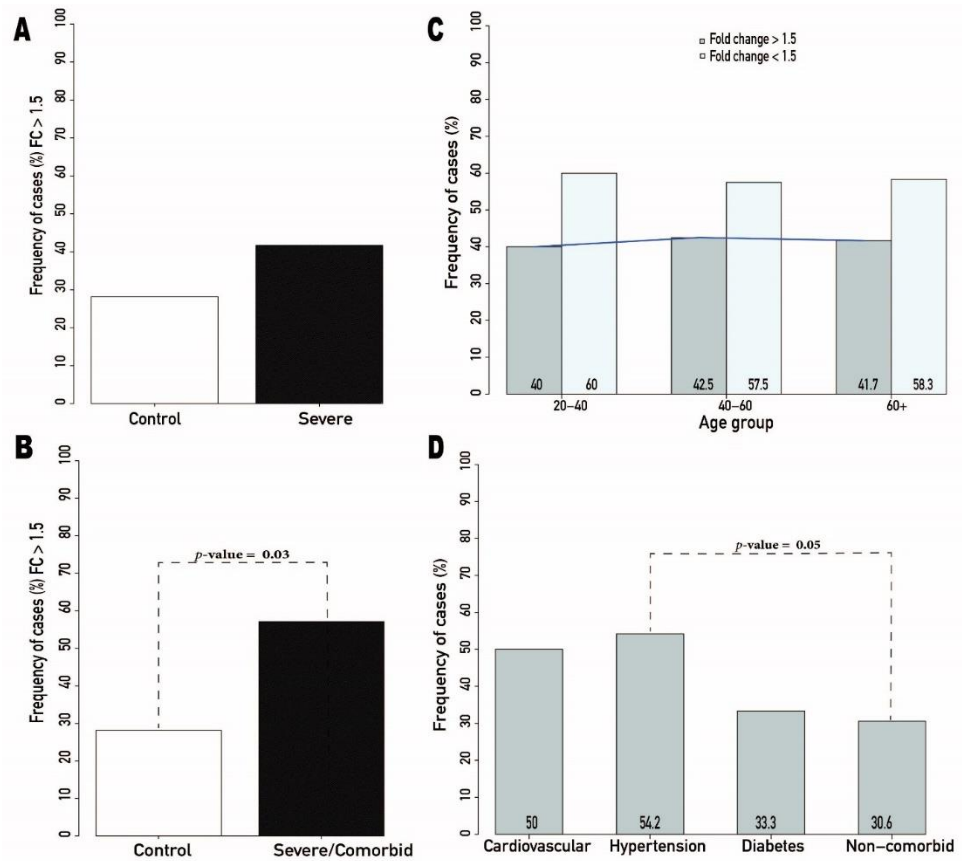

Figure S14. Frequency of cases with fold change greater than 1.5 of *FFAR2* gene. Related to Figure 4. A) control vs severe cases. B) control vs severe cases with comorbidity. C) different age group. D) different comorbidities.

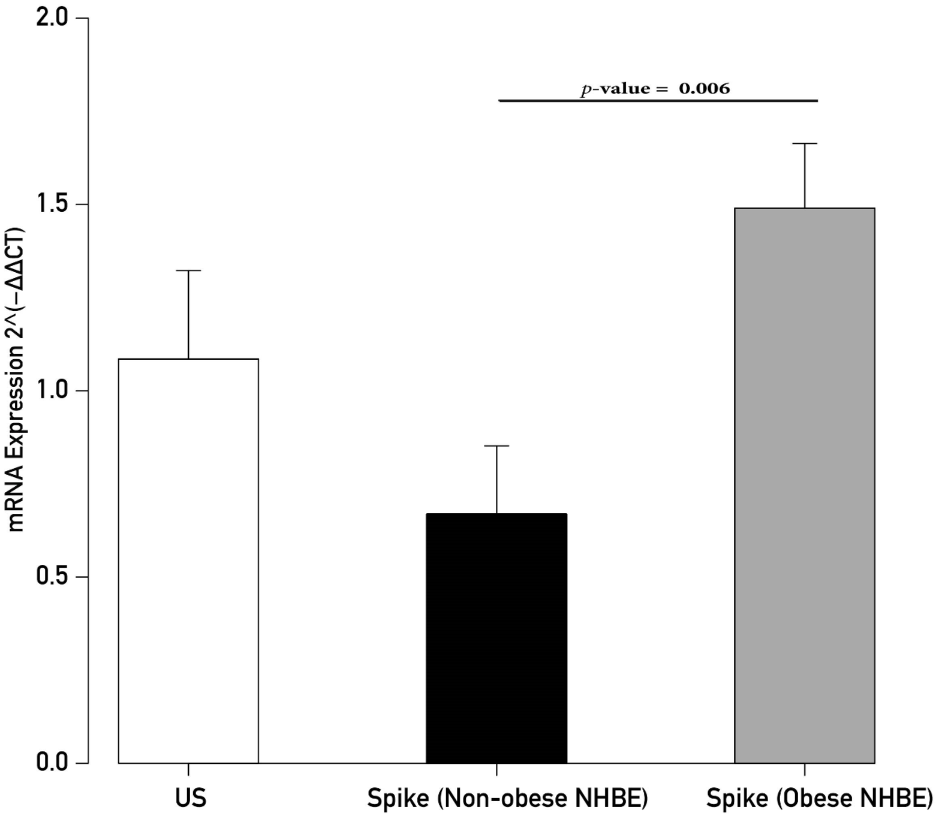

Figure S15. Expression of *FCGR3B* in non-obese and obese subject's normal human primary bronchial epithelial (NHBE) cells infected with or without spike protein. Related to Figure 4.

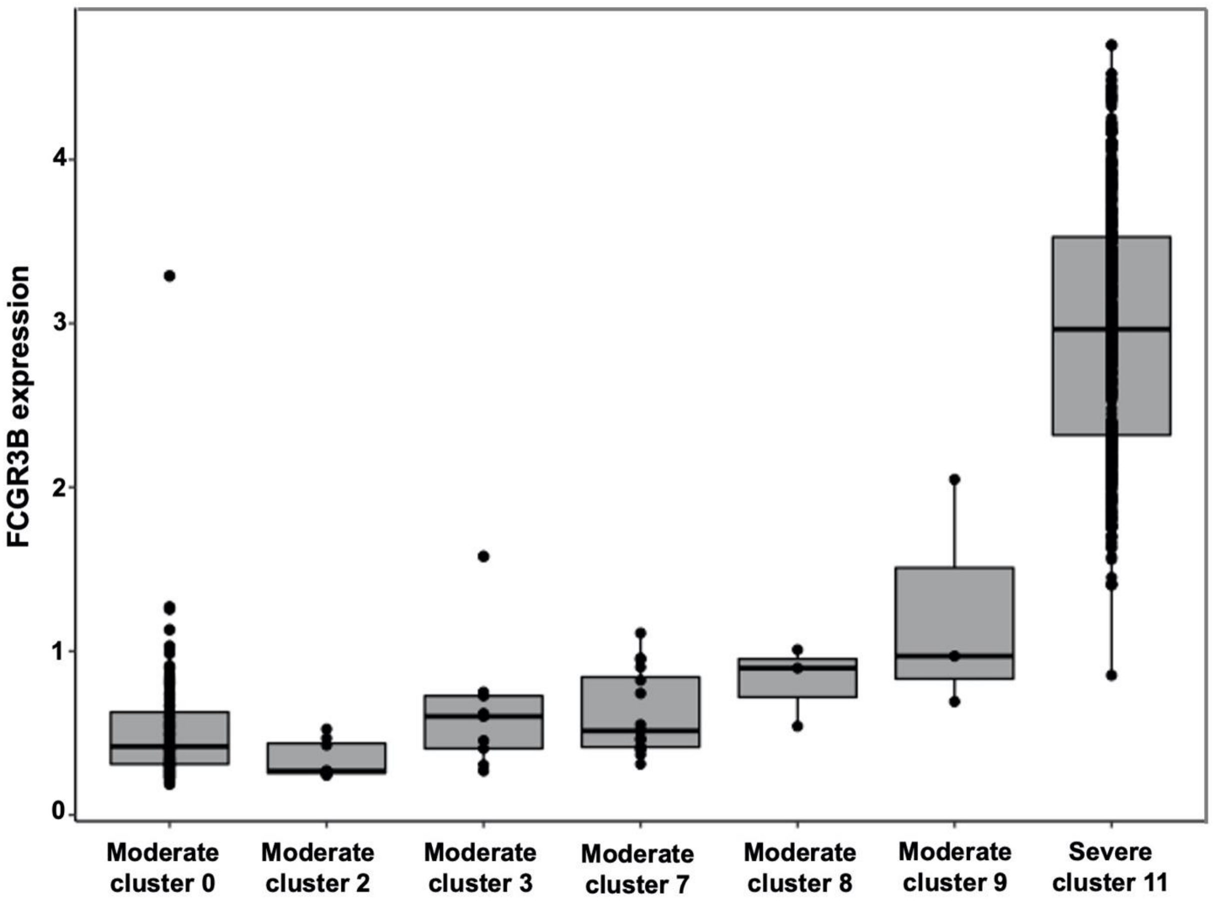

Figure S16. The expression of FCGR3B in CCL3L1 upregulated (Expression> Mean expression of CCL3L1 in all the 3-dataset – 0.885) cells for the moderate and severe data. Related to Figure 3. FCGR3B was expressed in 9%, 1%, 1%, 5%, 1%, 1% and 59% of the CCL3L1 upregulated cells in Moderate cluster 0, Moderate cluster 2, Moderate cluster 3, Moderate cluster 7, Moderate cluster 8, Moderate cluster 9 and Severe cluster 11, respectively.

Supplementary Tables:

Table S6. Data of non-obese and obese subjects. Related to Figure 4.

	Non-obese (BMI ≤ 30)	Obese (BMI ≥ 30)
N	3	2
Age, yr	46 ± 16.1	32 ± 22.6
BMI, kg/m2	28.9 ± 1.1	36.9 ± 4.2

Definition of abbreviation: BMI = body mass index
Values shown are mean ± SE

Table S7. Forward and reverse primers of *FCGR3B* and housekeeping gene used in monocyte experiment. Related to Figure 4.

Primer name	Oligo sequence (5' to 3')
FCGR3B Forward	GGAGAGTACAGGTGCCAGACAA
FCGR3B Reverse	CCTCAGGTGAATAGGGTCTTCC
GAPDH Forward	GAAGGTGAAGGTCGGAGT
GAPDH Reverse	GAAGATGGTGATGGGATTTC

Table S10: Table showing the average expression and percent expression per cluster for CCL3L1 and FCGR3B across the "severe COVID single transcriptome cell types". Related to Figure 3.

Severe Cluster Identity	Cluster No.	Cells expressed (%)		Average expression	
		CCL3L1	***FCG3RB***	***CCL3L1***	***FCG3RB***
Basal	0	51.33	10.40	7.06	0.48
Vessels	1	84.29	18.61	42.69	1.00
Vessels	2	67.25	10.97	10.72	0.25
Vessels	3	66.01	8.39	13.28	0.41
Dendritic	4	69.66	16.51	10.50	0.43
vessels	5	86.96	15.30	36.20	0.34
Ionocyte	6	39.80	9.01	7.23	0.47
Ionocyte	7	30.83	9.28	5.91	0.62
Dendritic cell	8	63.85	14.26	16.41	0.77
Basal	9	51.06	12.98	15.48	1.11
Ionocyte	10	36.14	10.13	4.24	0.29
Moam_CCL3L1	11	86.43	70.61	143.65	19.41
Alveolar epithelium	12	24.60	7.79	3.88	0.66
Plasma	13	13.22	2.71	3.67	0.12
Alveolar epithelium	14	13.45	6.16	1.08	0.22
Plasma	15	9.76	2.40	0.54	0.05
Moam_CCL3L18	16	54.49	21.35	3.92	0.36
Alveolar epithelium	17	14.89	7.16	1.15	0.22

Table 11: Table showing the average expression and percent expression per cluster for CCL3L1 and FCGR3B across the "moderate single transcriptome cell types". Related to Figure 3.

Moderate Cluster Identity	Cluster No.	Cells expressed (%)		Average expression	
		CCL3L1	*FCG3RB*	*CCL3L1*	*FCG3RB*
Serous	0	33.56	63.94	1.52	0.63
T cell	1	6.79	1.48	0.35	0.02
Myofibroblast	2	15.79	42.62	0.25	0.22
Serous	3	7.92	38.06	0.23	0.40
T cell	4	8.96	2.64	0.37	0.02
T cell	5	2.77	6.04	0.20	0.06
NA	6	14.56	19.27	0.84	0.16
Vessels	7	32.45	41.39	1.16	0.34
Vessels	8	12.27	28.16	0.65	0.45
TRAM	9	9.60	20.40	0.55	0.87
Anterior foregut endoderm	10	27.92	7.50	4.91	0.08
Alveolar epithelium	11	0.85	0.85	0	0.01
Club	12	0	0.99	0	0.01
Dendritic	13	2.04	0	0.15	0
Squamous corn epi	14	6.59	1.10	0.11	0.01
Cytotoxic t cell	15	3.08	0	0.04	0
B cells	16	0	1.85	0	0.04

Table S12: Table showing the average expression and percent expression per cluster for CCL3L1 and FCGR3B across the "control single transcriptome cell types". Related to Figure 3.

Control Cluster Identity	Cluster No.	Cells expressed (%)		Average expression	
		CCL3L1	*FCG3RB*	*CCL3L1*	*FCG3RB*
Brush cell	0	0.77	0.03	0.01	0
Serous	1	1.51	0.10	0.06	0
Serous	2	3.00	0.11	0.12	0
Serous	3	3.96	0.09	0.22	0
NA	4	5.10	0.22	0.15	0
Serous	5	12.26	0	0.91	0
Serous	6	2.07	0	0.20	0
TRAM	7	2.85	0.22	0.04	0
Club	8	0.37	0	0.02	0
Ciliated	9	0.13	0	0	0
anterior foregut endoderm	10	1.13	0	0.08	0
Moam_CCL18	11	0.20	0	0	0
Club	12	0.25	0	0	0
T cell	13	6.23	0	0.53	0
Cycling cell	14	2.00	0	0.05	0
Alveolar epithelium	15	0.49	0	0.05	0
Club	16	0	0	0	0
NA	17	1.00	0	0.01	0
Myeloid dendritic	18	5.26	0	0.27	0
